# Supplementary material for: Polar surface structure of oxide nanocrystals revealed with solid-state NMR spectroscopy
Source: Nat Commun. 2019 Nov 28;10:5420. doi: 10.1038/s41467-019-13424-7 (PMC6882792; doi:10.1038/s41467-019-13424-7)
Supplement: Supplementary file 1 — Supplementary Information [file 41467_2019_13424_MOESM1_ESM.pdf]

Supplementary Information for

Polar Surface Structure of Oxide Nanocrystals Revealed with Solid-State NMR Spectroscopy

Chen et al.

The Supplementary file includes:

Section A: Characterization of ceria nanocubes (XRD, TEM, XPS, RAMAN, surface area and composition)

(Supplementary Figures 1 – 4, Supplementary Note 1 and Supplementary Table 1)

Section B: ^{17}O and ^{1}H NMR data of NCs-$^{17}O_2$ and NCs-$H_2{}^{17}O$

(Supplementary Figures 5 – 7, Supplementary Tables 2 and 3)

Section C: DFT calculations of ^{17}O NMR parameters on O-t surfaces with different numbers of dissociated water molecules

(Supplementary Figures 8 – 23, Supplementary Tables 4 – 8)

Section D: Calculations of the adsorption site and energies of H_2O on CeO_4-t and O-t surface units

(Supplementary Figures 24 – 28, Supplementary Table 9)

Section E: DFT calculations of ^{17}O NMR parameters on CeO_4-t surfaces with different numbers of dissociated water molecules

(Supplementary Figures 29 – 40, Supplementary Note 2 and Supplementary Tables 10 – 13)

Section F: The determination of the ^{1}H δ_{ref} for M_2 and M_3

(Supplementary Figures 41 and 42)

Section G: ^{17}O direct DNP MATPASS NMR spectrum of NCs-$H_2{}^{17}O$

(Supplementary Figure 43)

Supplementary References

Section A: Characterization of ceria nanocubes (XRD, TEM, XPS, RAMAN, surface area and composition)

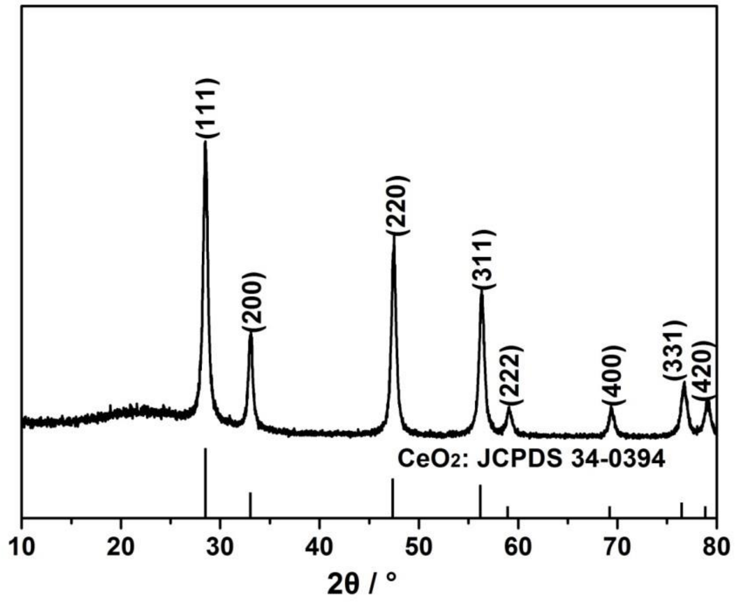

Supplementary Figure 1. XRD pattern of as-obtained ceria nanocubes.

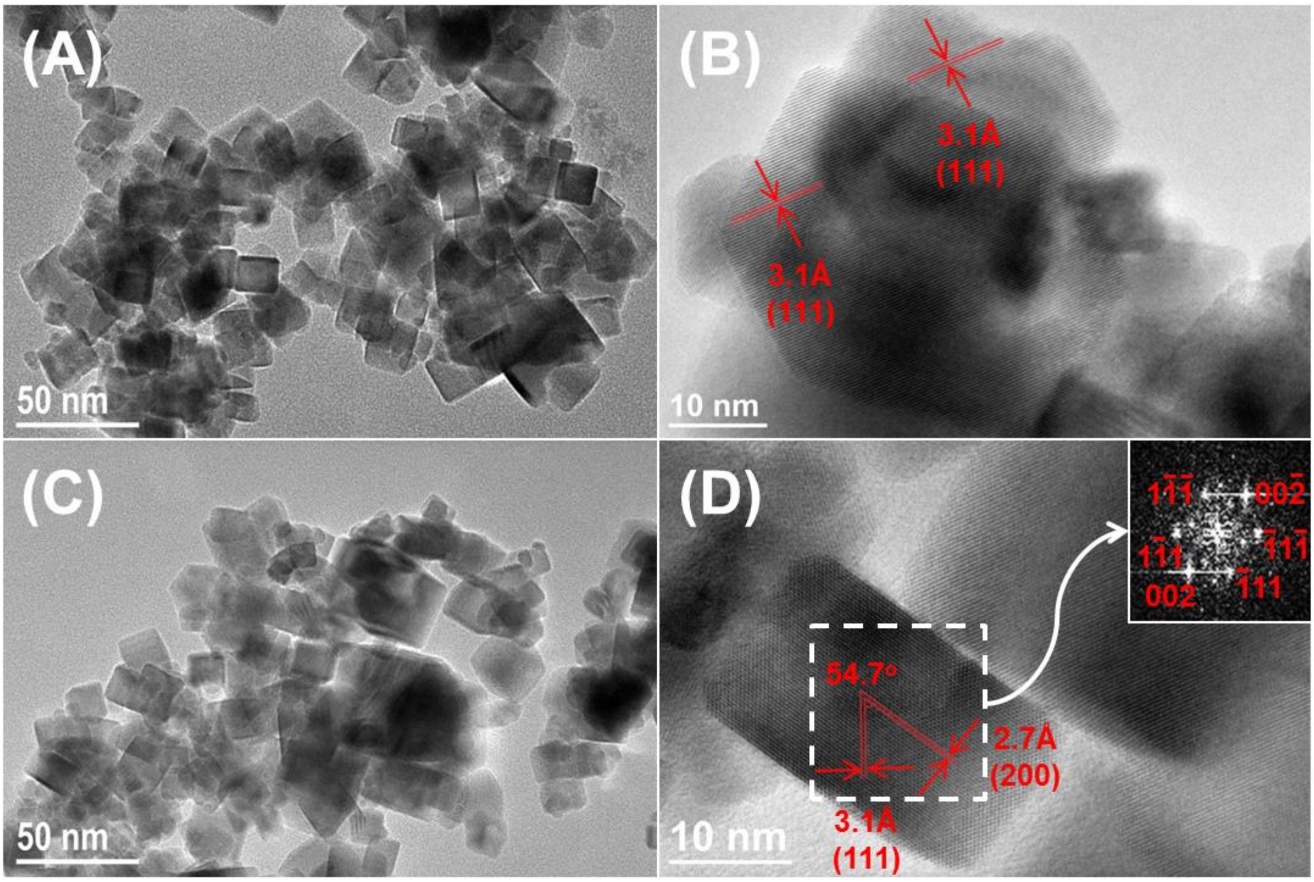

Supplementary Figure 2. HRTEM images of the ^{17}O-labeled ceria nanocubes. (A, B) NCs-$^{17}O_2$ and (C, D) NCs-$H_2{}^{17}O$.

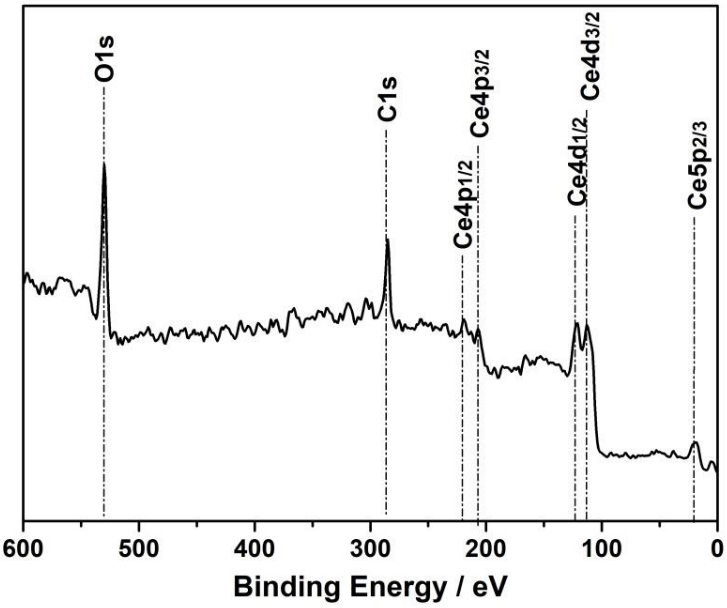

Supplementary Figure 3. XPS spectra of as-obtained ceria nanocubes.

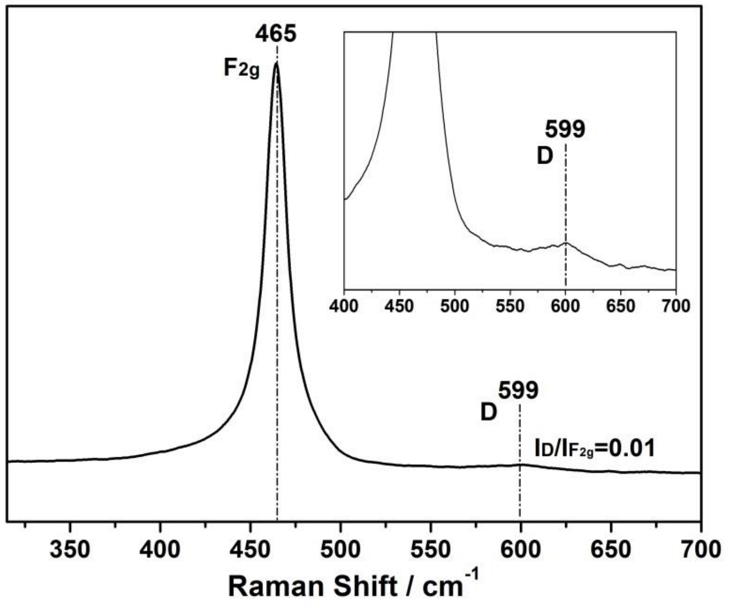

Supplementary Figure 4. Raman spectra (514 nm) of as-obtained ceria nanocubes.

Supplementary Note 1

Two bands were detected in the Raman spectrum of ceria nanocubes. The intense band at 465 cm^{-1} is assigned to the F_{2g} vibrational mode of the ceria fluorite-type structure,[1] while the weak band at 599 cm^{-1} represents oxygen vacancy sites.[2] Since the Raman spectrum excited with the 514 nm laser can only provide surface information for ceria, the intensity ratio of the bands at 599 and 465 cm^{-1} (I_D/I_{F2g}) is utilized to estimate the amount of oxygen vacancies at the surface.[3,4]

Supplementary Table 1. Some properties of the ceria nanocubes.

Sample	S_{BET} [a] / m^2 g^{-1}	Crystal Size[b] / nm	V_{Oxygen} [c] / %	Na Content[d] / wt %	N Content[e] / wt %
Nanocubes	53.3	18 – 40	< 1.0	< 0.1	< 0.1

[a] S_{BET} represents the Brunauer-Emmett-Teller (BET) specific surface area of ceria nanocubes;

[b] Crystal size was obtained by analyzing HRTEM images in Supplementary Figure 2 (A);

[c] The content of oxygen vacancies at the surface was extracted by the deconvolution of Raman spectrum in Supplementary Figure 4;

[d] Na content was measured by applying inductively coupled plasma mass spectrometry (ICP-MS);

[e] N content was obtained with a CHN-0-Rapid elemental analyzer.

Section B: ^{17}O and ^{1}H NMR data of NCs-$^{17}O_2$ and NCs-$H_2{}^{17}O$

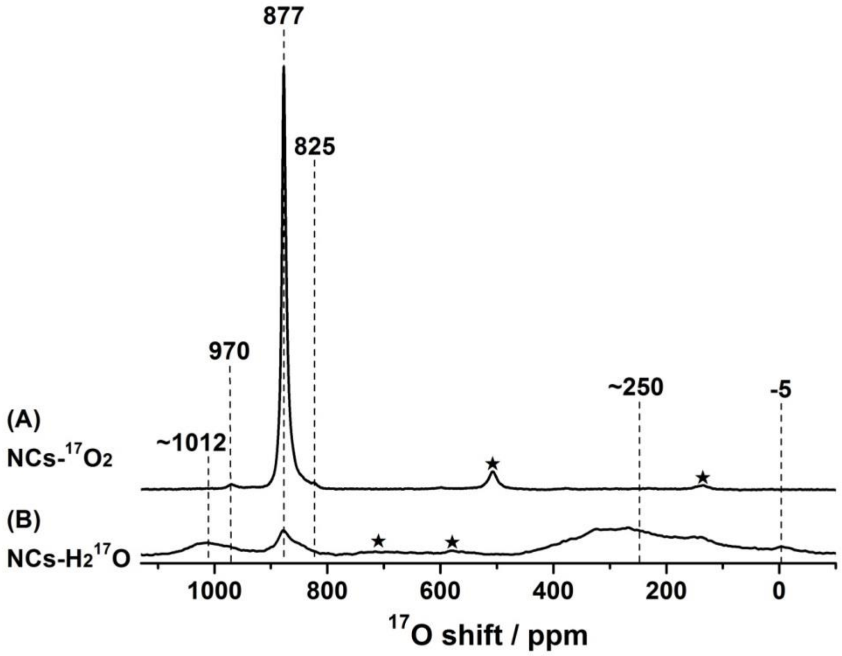

Supplementary Figure 5. Untruncated ^{17}O solid-state NMR spectra of the ^{17}O-labeled ceria nanocubes. (A) NCs-$^{17}O_2$ and (B) NCs-$H_2{}^{17}O$. Stars denote spinning sidebands.

Supplementary Table 2. The chemical shifts (δ_{iso}), peak widths and integrated intensities of the deconvoluted ^{1}H NMR signals.

	NCs-$^{17}O_2$			NCs-$H_2{}^{17}O$		
	δ_{iso} / ppm	Width / Hz	Integrated Intensity / %	δ_{iso} / ppm	Width / Hz	Integrated Intensity / %
-OH_T	2.5	350	6.3	2.5	350	5.6
H_2O	5.4	620	7.6	5.4	600	5.8
-OH_B	4.1	560	3.8	5.0	1060	16.6
	6.8	530	4.9	6.3	660	4.6
	8.0	660	60.3	8.0	880	25.1
	9.7	1260	17.1	9.4	960	23.0
	—	—	—	11.4	1920	19.3

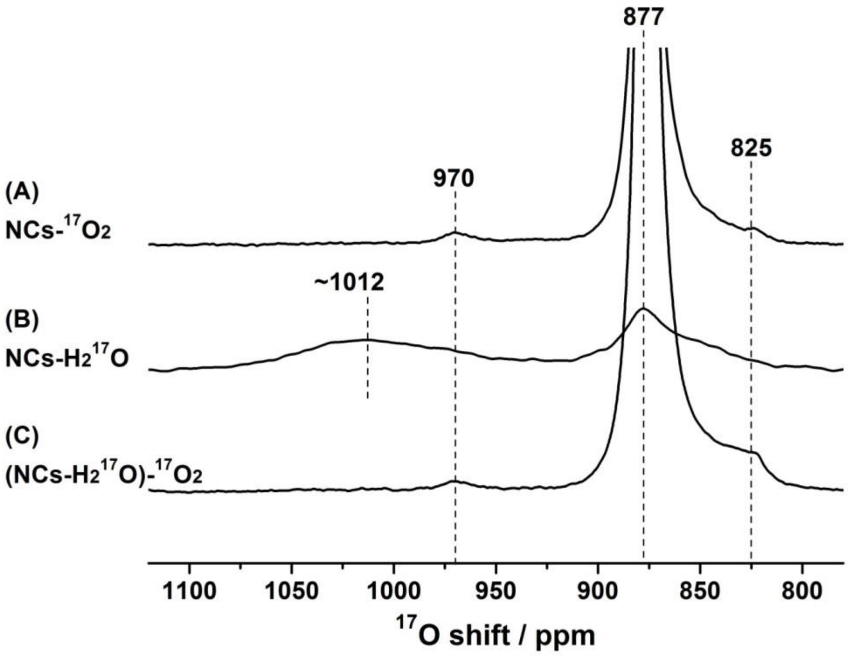

Supplementary Figure 6. ^{17}O solid-state NMR spectra of the ^{17}O-labeled ceria nanocubes. (A) NCs-$^{17}O_2$, (B) NCs-$H_2{}^{17}O$ and (C) NCs-$H_2{}^{17}O$ after re-enrichment with $^{17}O_2$ ((NCs-$H_2{}^{17}O$)-$^{17}O_2$).

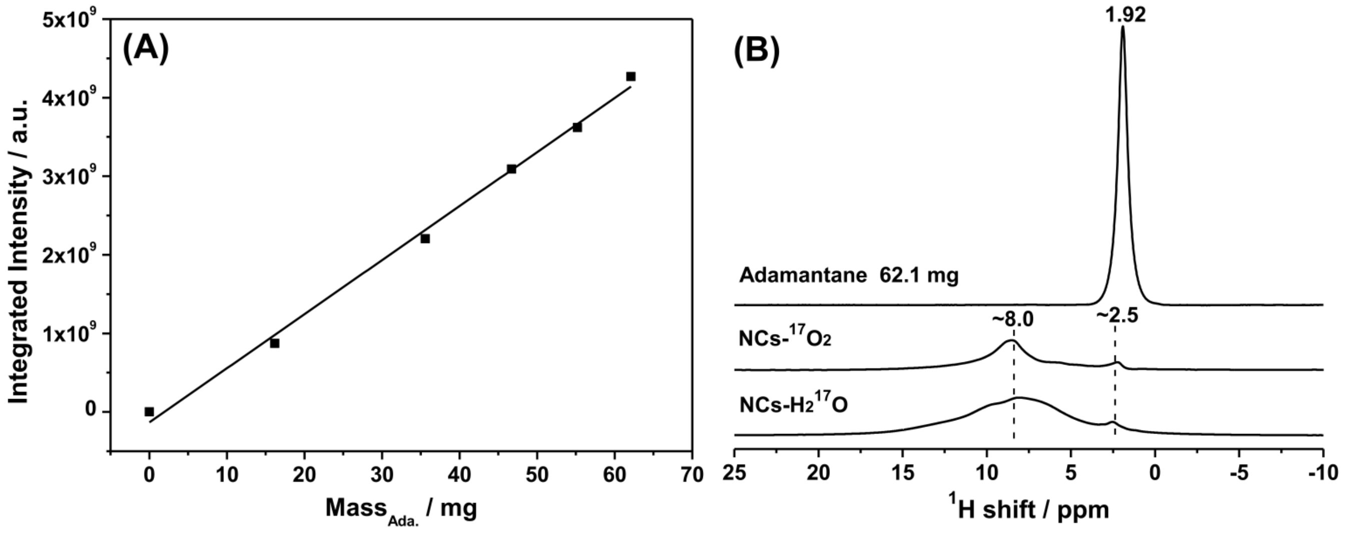

Supplementary Figure 7. Interpolation method for determining the amount of water in ceria nanocubes from quantitative ^{1}H NMR, using adamantane (ada.) as a reference material.[6] (A) The integrated intensity of the ^{1}H resonance as a function of the mass of adamantane packed in the center part of the rotor. The integral range is set to -10 – 25 ppm. The number of scans acquired is 16 and the spinning speed is 12 kHz. A long recycle delay of 8 s is used to ensure quantitative measurement. The content of hydrogen is determined from the mass of adamantane. The linear correlation between the integrated intensity (y) and the mass of adamantane (x/mg) is y=-1.8×10^{8}+6.9×10^{7}x. (B) Conventional ^{1}H solid-state NMR spectra of adamantane, NCs-$^{17}O_2$ and NCs-$H_2{}^{17}O$. The same integral range, quantitative recycle delay and spinning speed are used. The number of scans acquired is 192.

Supplementary Table 3. The concentration of adsorbed water molecule in NCs-$^{17}O_2$ and NCs- $H_2{}^{17}O$.[a]

Sample	Mass / mg	Integrated Intensity from 192 Scans / a.u.	Amount of Hydrogen / mol g^{-1}	Concentration of H_2O[b] / Surface Unit^{-1}
Ada.	62.1	4.9×10^{10}	1.2×10^{-1}	—
NCs-$^{17}O_2$	122.1	6.6×10^{8}	7.9×10^{-4}	2.6
NCs- $H_2{}^{17}O$	120.8	9.1×10^{8}	1.1×10^{-3}	3.7

[a] The concentration of adsorbed water molecules is determined by the interpolation method using adamantane as a reference material;[6]

[b] The concentration of adsorbed water molecule is acquired from the following formula:

H_2O (Surface Unit^{-1}) = (Amount of Hydrogen (mol g^{-1})) $\times$ (N_A) / (S_{BET} (m^2 g^{-1})) / (S_{Unit} (m^2)) / 2, where N_A represents the Avogadro constant (6.02×10^{23} mol^{-1}), S_{BET} = 53.3 m^2 g^{-1} is the sample surface area from BET, and S_{Unit} denotes the surface area of one Surface Unit (7.7 Å $\times$ 7.7 Å = 59.3 Å^2, Figure 3).

Section C: DFT calculations of ^{17}O NMR parameters on O-t surfaces with different numbers of dissociated water molecules

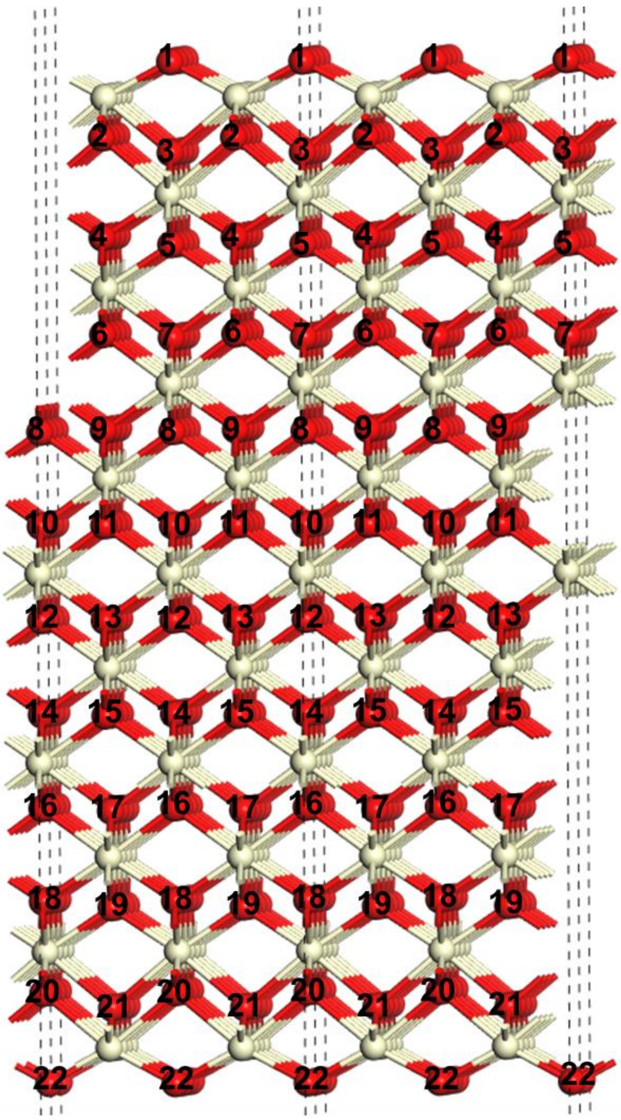

Supplementary Figure 8. Calculated structure of clean O-t ceria (100) surface model, which contains 12 layers of oxygen and 11 layers of cerium ions. Red and off-white spheres represent oxygen and cerium ions, respectively. Oxygen ions with the same chemical environment (thus the same NMR parameters) are labeled with the same number.

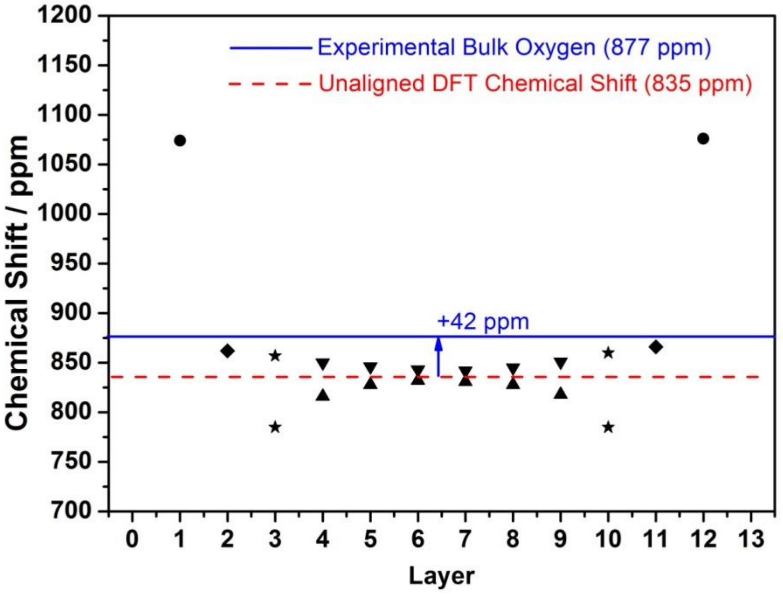

Supplementary Figure 9. The determination of the ^{17}O reference chemical shift (δ_{ref}) from the clean O-t model. Spheres, diamonds, stars and triangles represent the unaligned DFT chemical shifts of oxygen ions at the top/bottom 1st, 2nd and 3rd surface layers and the inner part of the structure ("bulk"). The red dashed line shows the average value of the unaligned DFT chemical shifts of the oxygen ions in the "bulk".

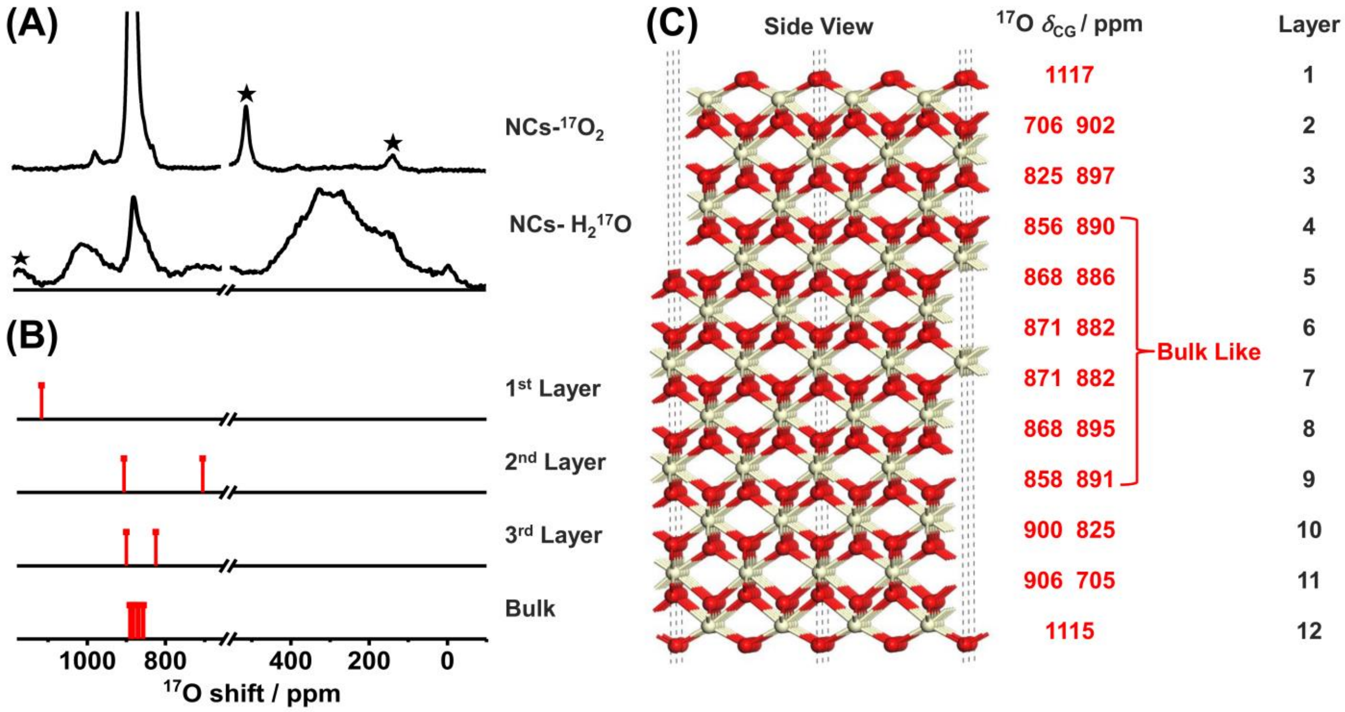

Supplementary Figure 10. ^{17}O NMR spectra, calculated ^{17}O NMR shifts and the clean O-t structure model of ceria. (A) ^{17}O solid-state NMR spectra of ceria NCs-$^{17}O_2$ and NCs-$H_2{}^{17}O$. (B) The summarized ^{17}O NMR shifts (δ_{CG}s) predicted in the clean O-t model shown in (C). (C) Model used in DFT calculations with calculated shifts (δ_{CG}s) for oxygen ions (labeled in Supplementary Figure 8) and the layer numbers shown on the right. The calculated ^{17}O NMR parameters for each oxygen ions are shown in Supplementary Table 4.

Supplementary Table 4. Calculated ^{17}O NMR parameters for oxygen ions in the O-t model. Isotropic chemical shifts (δ_{iso}), quadrupolar parameters (C_Q and η) and calculated NMR shifts (center of gravity, δ_{CG}) for each resonance are shown. δ_{CG} is calculated according to Lippmaa[7] at 9.4 T including the second order quadrupolar shift. The corresponding structure is presented in Supplementary Figure 8.

O_{ion} No.	δ_{iso} / ppm	C_Q / MHz	η	δ_{CG} / ppm	Assignment
1	1118	0.74	0.69	1117	1st Layer O_{2C}
2	706	0.11	0.86	706	2nd Layer O_{4C}
3	902	0.11	0.75	902	2nd Layer O_{4C}
4	825	0.11	0.09	825	3rd Layer O_{4C}
5	897	0.11	0.07	897	3rd Layer O_{4C}
6	856	0.12	0.03	856	Bulk Like
7	890	0.12	0.03	890	Bulk Like
8	886	0.11	0.03	886	Bulk Like
9	868	0.11	0.01	868	Bulk Like
10	883	0.11	0.01	883	Bulk Like
11	872	0.12	0.05	872	Bulk Like
12	882	0.10	0.01	882	Bulk Like
13	871	0.11	0.18	871	Bulk Like
14	885	0.10	0.12	885	Bulk Like
15	868	0.10	0.06	868	Bulk Like
16	891	0.11	0.09	891	Bulk Like
17	858	0.10	0.05	858	Bulk Like
18	900	0.11	0.07	900	10th Layer O_{4C}
19	825	0.11	0.07	825	10th Layer O_{4C}
20	906	0.11	0.08	906	11th Layer O_{4C}
21	705	0.11	0.86	705	11th Layer O_{4C}
22	1116	0.73	0.66	1115	12th Layer O_{2C}

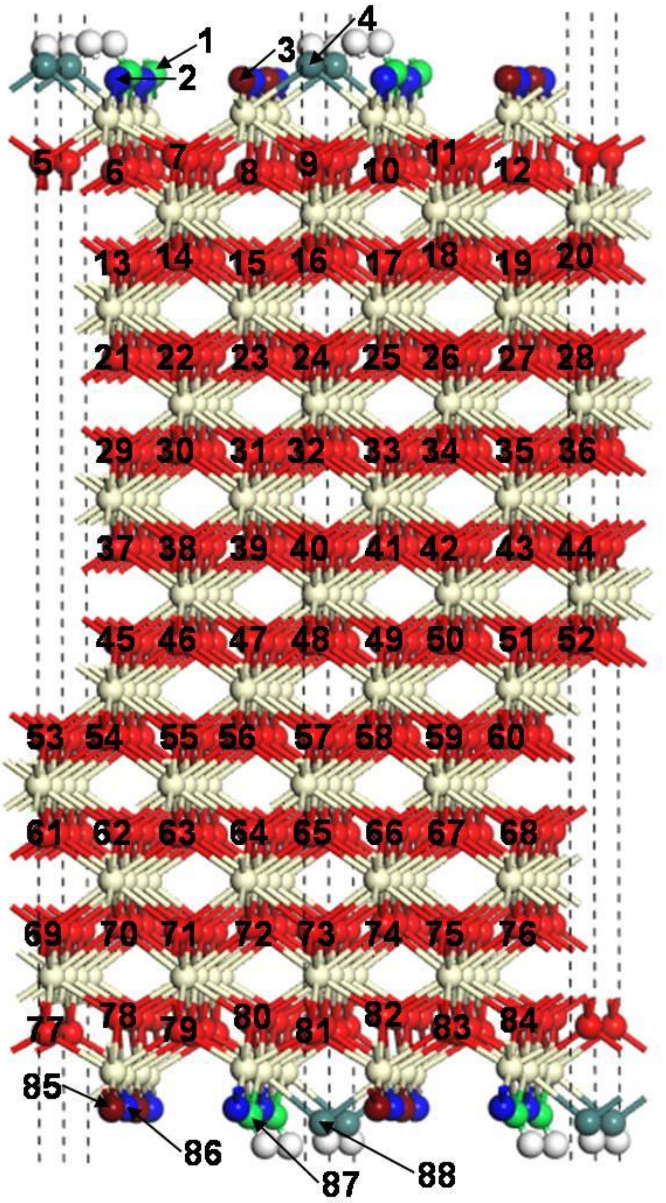

Supplementary Figure 11. Calculated structure of the O-t model with one H_2O molecule dissociatively adsorbed on each surface unit. Oxygen ions with the same chemical environment (thus the same NMR parameters) are labeled with the same number. Red, off-white and white spheres represent internal oxygen, cerium and hydrogen ions, respectively. Surface oxygen groups with different chemical shifts are in different colors.

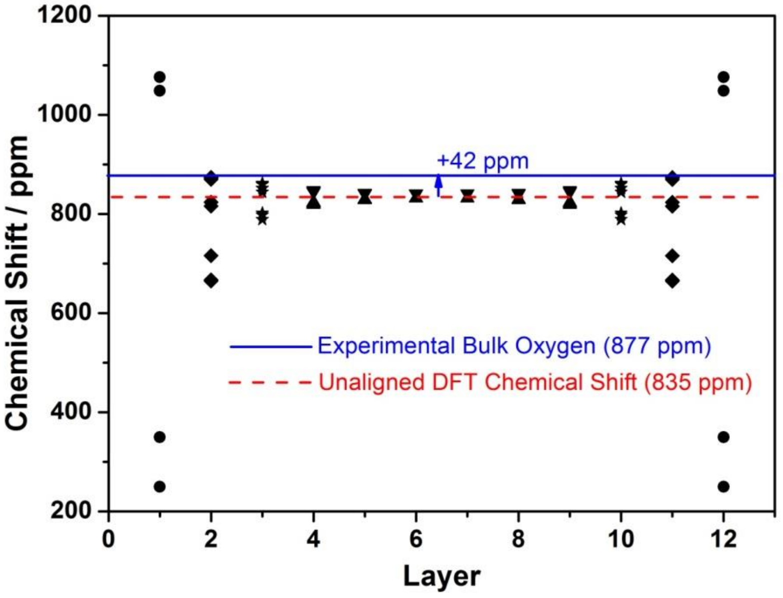

Supplementary Figure 12. The determination of the ^{17}O reference chemical shift (δ_{ref}) from the O-t model with one H_2O molecule dissociatively adsorbed on each surface unit. Spheres, diamonds, stars and triangles represent the unaligned DFT chemical shifts of oxygen ions at the top/bottom 1st, 2nd and 3rd surface layers and the inner part of the structure ("bulk"). The red dashed line shows the average value of the unaligned DFT chemical shifts of the oxygen ions in the "bulk".

Supplementary Table 5. Calculated ^{17}O NMR parameters for oxygen ions in the O-t model with one H_2O molecule dissociatively adsorbed on each surface unit. Isotropic chemical shifts (δ_{iso}), quadrupolar parameters (C_Q and η) and calculated NMR shifts (center of gravity, δ_{CG}) for each resonance are shown. The corresponding structure is presented in Supplementary Figure 11.

O_{ion} No.	δ_{iso} / ppm	C_Q / MHz	η	δ_{CG} / ppm	Assignment
1	292	6.96	0.23	191	Surface -OH
2	1118	0.79	0.69	1117	1st Layer O_{2C}
3	1091	0.71	0.41	1090	1st Layer O_{2C}
4	392	6.53	0.10	305	Surface -OH
5	758	0.47	0.86	757	2nd Layer O_{4C}
6	866	0.48	0.52	865	2nd Layer O_{4C}
7	858	0.84	0.43	856	2nd Layer O_{4C}
8	912	0.57	0.46	911	2nd Layer O_{4C}
9	709	0.88	0.53	707	2nd Layer O_{4C}
10	915	0.68	0.54	914	2nd Layer O_{4C}
11	707	0.64	0.65	706	2nd Layer O_{4C}
12	916	0.57	0.62	915	2nd Layer O_{4C}
13	904	0.09	0.28	904	3rd Layer O_{4C}
14	840	0.41	0.71	840	3rd Layer O_{4C}
15	886	0.09	0.18	886	3rd Layer O_{4C}
16	831	0.29	0.58	831	3rd Layer O_{4C}
17	902	0.21	0.31	902	3rd Layer O_{4C}
18	844	0.29	0.82	844	3rd Layer O_{4C}
19	894	0.24	0.90	894	3rd Layer O_{4C}
20	844	0.36	0.57	844	3rd Layer O_{4C}
21	888	0.13	0.34	888	Bulk Like
22	864	0.19	0.90	864	Bulk Like
23	890	0.13	0.82	890	Bulk Like
24	861	0.14	0.75	861	Bulk Like
25	890	0.14	0.40	890	Bulk Like
26	863	0.20	0.92	863	Bulk Like
27	887	0.11	0.35	887	Bulk Like
28	866	0.18	0.95	866	Bulk Like
29	884	0.09	0.81	884	Bulk Like
30	871	0.13	0.73	871	Bulk Like
31	884	0.09	0.52	884	Bulk Like
32	872	0.13	0.65	872	Bulk Like
33	883	0.09	0.86	883	Bulk Like
34	871	0.14	0.73	871	Bulk Like
35	883	0.09	0.49	883	Bulk Like
36	872	0.14	0.73	872	Bulk Like
37	883	0.09	0.39	883	Bulk Like
38	874	0.13	0.33	874	Bulk Like
39	883	0.08	0.51	883	Bulk Like
40	874	0.13	0.32	874	Bulk Like
41	883	0.09	0.40	883	Bulk Like
42	874	0.13	0.37	874	Bulk Like
43	883	0.09	0.45	883	Bulk Like

44	874	0.13	0.38	874	Bulk Like
45	884	0.09	0.18	884	Bulk Like
46	873	0.14	0.12	873	Bulk Like
47	884	0.09	0.14	884	Bulk Like
48	873	0.14	0.15	873	Bulk Like
49	884	0.09	0.28	884	Bulk Like
50	873	0.14	0.09	873	Bulk Like
51	884	0.09	0.17	884	Bulk Like
52	873	0.14	0.09	873	Bulk Like
53	872	0.15	0.66	872	Bulk Like
54	887	0.08	0.62	887	Bulk Like
55	870	0.15	0.65	870	Bulk Like
56	887	0.09	0.87	887	Bulk Like
57	872	0.14	0.49	872	Bulk Like
58	885	0.08	0.62	885	Bulk Like
59	870	0.15	0.62	870	Bulk Like
60	885	0.09	0.76	885	Bulk Like
61	865	0.18	0.98	865	Bulk Like
62	888	0.11	0.40	888	Bulk Like
63	862	0.21	0.88	862	Bulk Like
64	891	0.14	0.39	891	Bulk Like
65	860	0.17	0.78	860	Bulk Like
66	891	0.13	0.86	891	Bulk Like
67	864	0.20	0.89	864	Bulk Like
68	888	0.13	0.33	888	Bulk Like
69	844	0.36	0.54	844	10th Layer O_{4C}
70	894	0.24	0.85	894	10th Layer O_{4C}
71	844	0.30	0.78	844	10th Layer O_{4C}
72	902	0.21	0.30	902	10th Layer O_{4C}
73	832	0.29	0.59	832	10th Layer O_{4C}
74	886	0.22	0.49	886	10th Layer O_{4C}
75	839	0.42	0.69	839	10th Layer O_{4C}
76	903	0.28	0.44	903	10th Layer O_{4C}
77	709	0.88	0.54	707	11th Layer O_{4C}
78	916	0.58	0.61	915	11th Layer O_{4C}
79	706	0.63	0.64	705	11th Layer O_{4C}
80	914	0.68	0.54	913	11th Layer O_{4C}
81	759	0.48	0.86	758	11th Layer O_{4C}
82	911	0.57	0.45	910	11th Layer O_{4C}
83	859	0.86	0.44	857	11th Layer O_{4C}
84	866	0.48	0.52	865	11th Layer O_{4C}
85	1091	0.71	0.63	1091	12th Layer O_{2C}
86	1118	0.37	0.29	1118	12th Layer O_{2C}
87	392	6.79	0.70	305	Surface -OH
88	292	6.96	0.23	191	Surface -OH

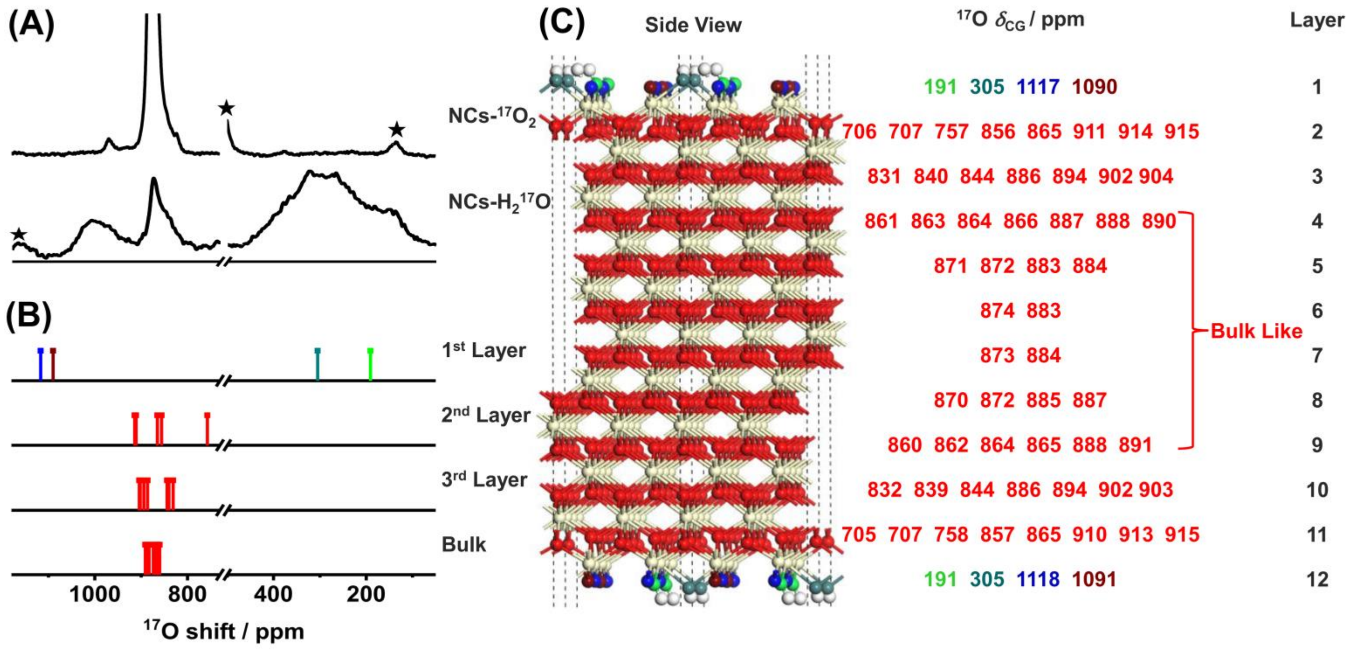

Supplementary Figure 13. ^{17}O NMR spectra, calculated ^{17}O NMR shifts and the O-t structure model with one H_2O molecule dissociatively adsorbed on each surface unit. (A) ^{17}O solid-state NMR spectra of ceria NCs-$^{17}O_2$ and NCs-$H_2{}^{17}O$. (B) The summarized ^{17}O NMR shifts (δ_{CG}s) predicted in the O-t model with one H_2O molecule dissociatively adsorbed on each surface unit shown in (C). (C) Model used in DFT calculations with calculated NMR shifts (δ_{CG}s) for oxygen ions (labeled in Supplementary Figure 11) and the layer number shown on the right. The calculated ^{17}O NMR parameters for each oxygen ion are shown in Supplementary Table 5.

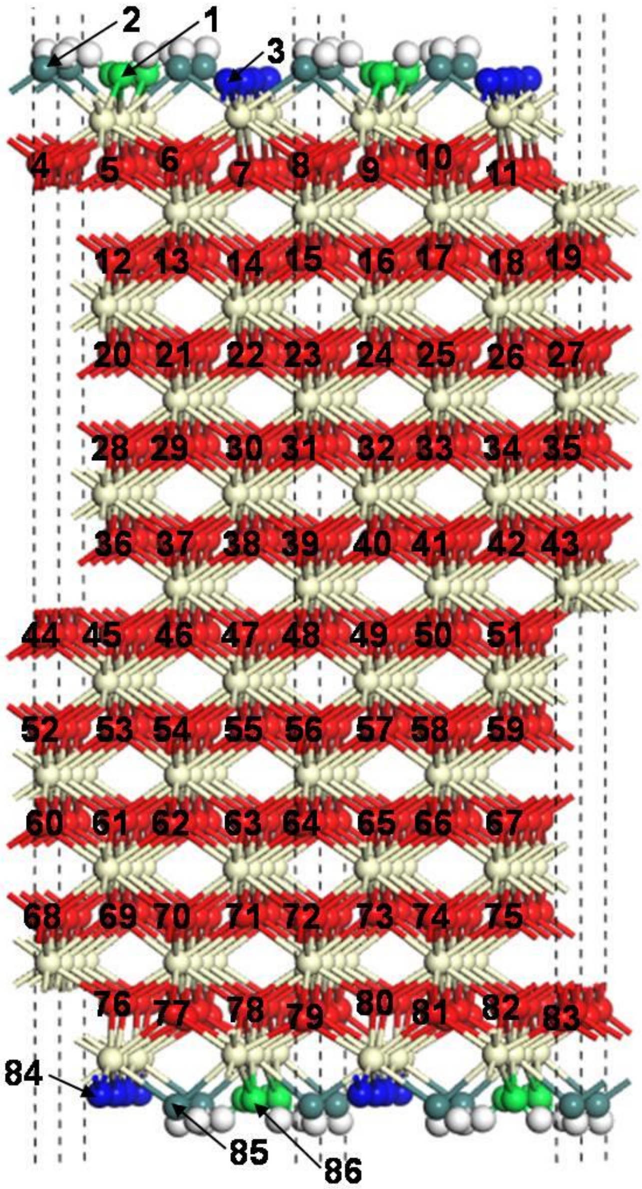

Supplementary Figure 14. Calculated structure of the O-t model with two H_2O molecules dissociatively adsorbed on each surface unit. Oxygen ions with the same chemical environment (thus the same NMR parameters) are labeled with the same number. Red, off-white and white spheres represent internal oxygen, cerium and hydrogen ions, respectively. Surface oxygen groups with different chemical shifts are in different colors.

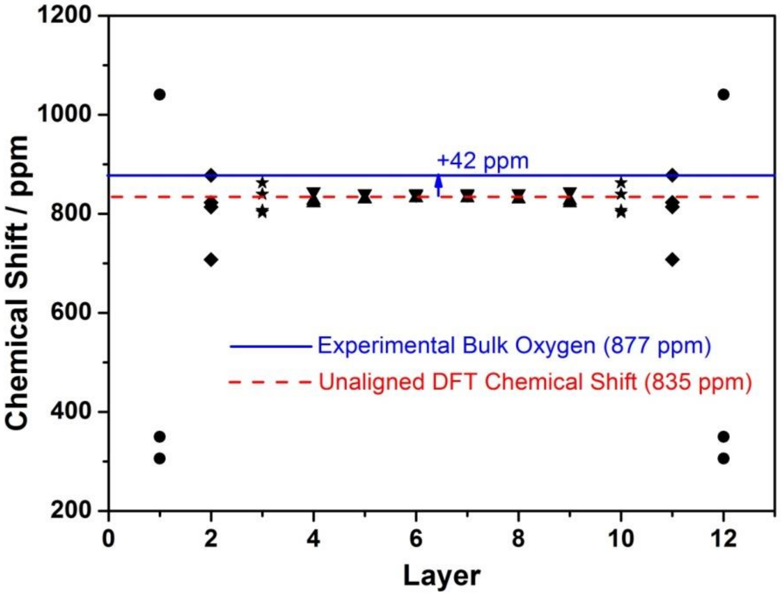

Supplementary Figure 15. The determination of the ^{17}O reference chemical shift (δ_{ref}) from the O-t model with two H_2O molecules dissociatively adsorbed on each surface unit. Spheres, diamonds, stars and triangles represent the unaligned DFT chemical shifts of oxygen ions at the top/bottom 1st, 2nd and 3rd surface layers and the inner part of the structure ("bulk"). The red dashed line shows the average value of the unaligned DFT chemical shifts of the oxygen ions in the "bulk".

Supplementary Table 6. Calculated ^{17}O NMR parameters for oxygen ions in the O-t model with two H_2O molecules dissociatively adsorbed on each surface unit. Isotropic chemical shifts (δ_{iso}), quadrupolar parameters (C_Q and η) and calculated NMR shifts (center of gravity, δ_{CG}) for each resonance are shown. The corresponding structure is presented in Supplementary Figure 14.

O_{ion} No.	δ_{iso} / ppm	C_Q / MHz	η	δ_{CG} / ppm	Assignment
1	348	6.19	0.25	268	Surface -OH
2	392	6.49	0.04	306	Surface -OH
3	1083	0.77	0.52	1082	1st Layer O_{2C}
4	750	0.43	0.63	750	2nd Layer O_{4C}
5	865	0.45	0.54	865	2nd Layer O_{4C}
6	856	0.90	0.39	854	2nd Layer O_{4C}
7	920	0.44	0.22	920	2nd Layer O_{4C}
8	856	0.89	0.39	854	2nd Layer O_{4C}
9	865	0.45	0.55	865	2nd Layer O_{4C}
10	750	0.43	0.63	750	2nd Layer O_{4C}
11	920	0.44	0.23	920	2nd Layer O_{4C}
12	905	0.18	0.60	905	3rd Layer O_{4C}
13	849	0.37	0.67	849	3rd Layer O_{4C}
14	882	0.19	0.83	882	3rd Layer O_{4C}
15	845	0.21	0.98	845	3rd Layer O_{4C}
16	905	0.18	0.61	905	3rd Layer O_{4C}
17	845	0.21	0.99	845	3rd Layer O_{4C}
18	882	0.18	0.82	882	3rd Layer O_{4C}
19	849	0.37	0.67	849	3rd Layer O_{4C}
20	887	0.12	0.66	887	Bulk Like
21	869	0.18	0.82	869	Bulk Like
22	887	0.10	0.52	887	Bulk Like
23	864	0.12	0.93	864	Bulk Like
24	887	0.12	0.66	887	Bulk Like
25	864	0.16	0.93	864	Bulk Like
26	887	0.10	0.49	887	Bulk Like
27	869	0.18	0.82	869	Bulk Like
28	883	0.10	0.56	883	Bulk Like
29	872	0.14	0.58	872	Bulk Like
30	873	0.08	0.66	873	Bulk Like
31	872	0.13	0.48	872	Bulk Like
32	873	0.10	0.58	873	Bulk Like
33	872	0.13	0.48	872	Bulk Like
34	873	0.08	0.65	873	Bulk Like
35	872	0.14	0.57	872	Bulk Like
36	881	0.10	0.17	881	Bulk Like
37	874	0.13	0.20	874	Bulk Like
38	883	0.09	0.30	883	Bulk Like
39	874	0.13	0.20	874	Bulk Like
40	881	0.10	0.17	881	Bulk Like
41	874	0.13	0.21	874	Bulk Like
42	883	0.09	0.31	883	Bulk Like
43	874	0.13	0.20	874	Bulk Like

44	874	0.13	0.17	874	Bulk Like
45	883	0.09	0.23	883	Bulk Like
46	874	0.13	0.15	874	Bulk Like
47	881	0.09	0.12	881	Bulk Like
48	874	0.13	0.17	874	Bulk Like
49	883	0.13	0.48	883	Bulk Like
50	874	0.13	0.16	874	Bulk Like
51	881	0.09	0.12	881	Bulk Like
52	872	0.14	0.59	872	Bulk Like
53	883	0.08	0.66	883	Bulk Like
54	872	0.13	0.49	872	Bulk Like
55	883	0.10	0.56	883	Bulk Like
56	872	0.13	0.50	872	Bulk Like
57	883	0.08	0.64	883	Bulk Like
58	872	0.14	0.60	872	Bulk Like
59	883	0.10	0.57	883	Bulk Like
60	869	0.17	0.89	869	Bulk Like
61	886	0.10	0.66	886	Bulk Like
62	863	0.16	0.92	863	Bulk Like
63	886	0.12	0.62	886	Bulk Like
64	863	0.16	0.87	863	Bulk Like
65	886	0.10	0.66	886	Bulk Like
66	869	0.18	0.85	869	Bulk Like
67	886	0.45	0.54	886	Bulk Like
68	848	0.21	0.97	848	10th Layer O_{4C}
69	882	0.19	0.75	882	10th Layer O_{4C}
70	845	0.21	0.97	845	10th Layer O_{4C}
71	904	0.18	0.64	904	10th Layer O_{4C}
72	845	0.21	0.99	845	10th Layer O_{4C}
73	882	0.19	0.75	882	10th Layer O_{4C}
74	848	0.36	0.64	848	10th Layer O_{4C}
75	904	0.18	0.66	904	10th Layer O_{4C}
76	920	0.44	0.23	920	11th Layer O_{4C}
77	750	0.44	0.63	750	11th Layer O_{4C}
78	865	0.44	0.56	865	11th Layer O_{4C}
79	856	0.90	0.38	854	11th Layer O_{4C}
80	920	0.44	0.23	920	11th Layer O_{4C}
81	856	0.89	0.39	854	11th Layer O_{4C}
82	865	0.45	0.55	865	11th Layer O_{4C}
83	750	0.43	0.62	750	11th Layer O_{4C}
84	1083	0.76	0.53	1082	12th Layer O_{2C}
85	392	6.47	0.04	306	Surface -OH
86	348	6.19	0.25	268	Surface -OH

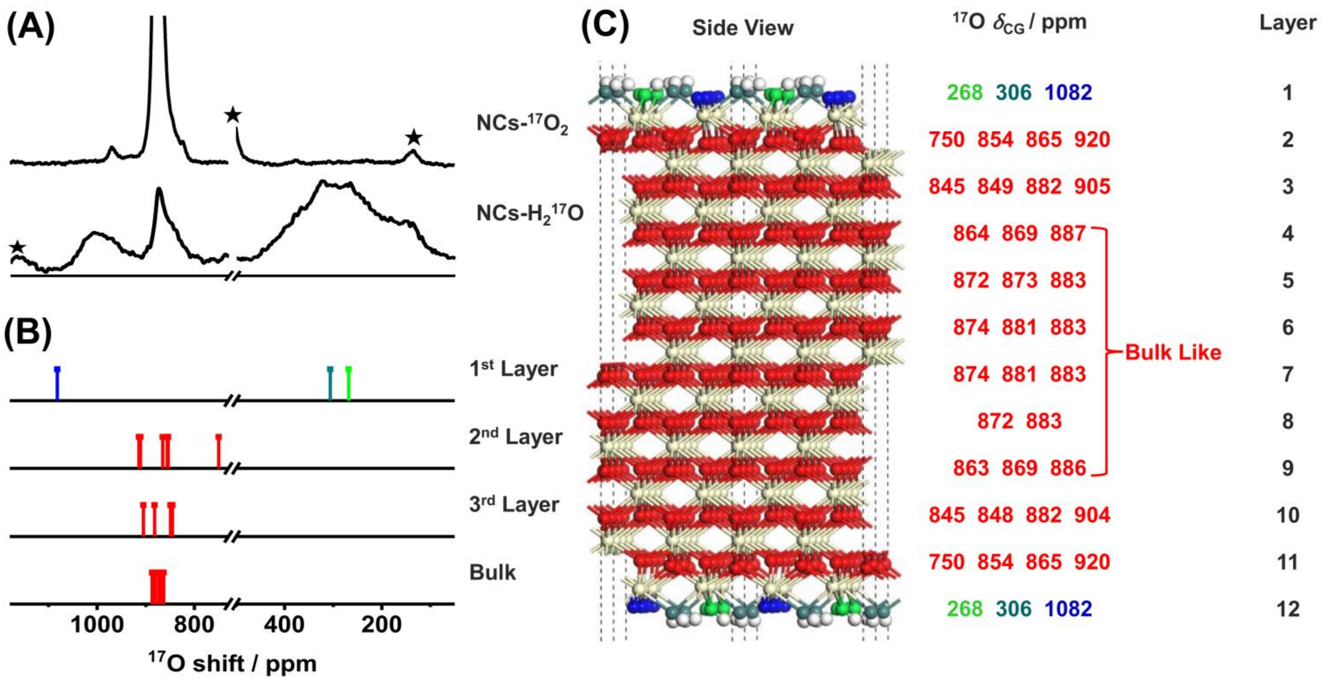

Supplementary Figure 16. ^{17}O NMR spectra, calculated ^{17}O NMR shifts and the O-t structure model with two H_2O molecules dissociatively adsorbed on each surface unit. (A) ^{17}O solid-state NMR spectra of ceria NCs-$^{17}O_2$ and NCs-$H_2{}^{17}O$. (B) The summarized ^{17}O NMR shifts (δ_{CG}s) predicted in the O-t model with two H_2O molecules dissociatively adsorbed on each surface unit shown in (C). (C) Model used in DFT calculations with calculated shifts (δ_{CG}s) for oxygen ions (labeled in Supplementary Figure 14) and the layer number shown on the right. The calculated ^{17}O NMR parameters for each oxygen ion are shown in Supplementary Table 6.

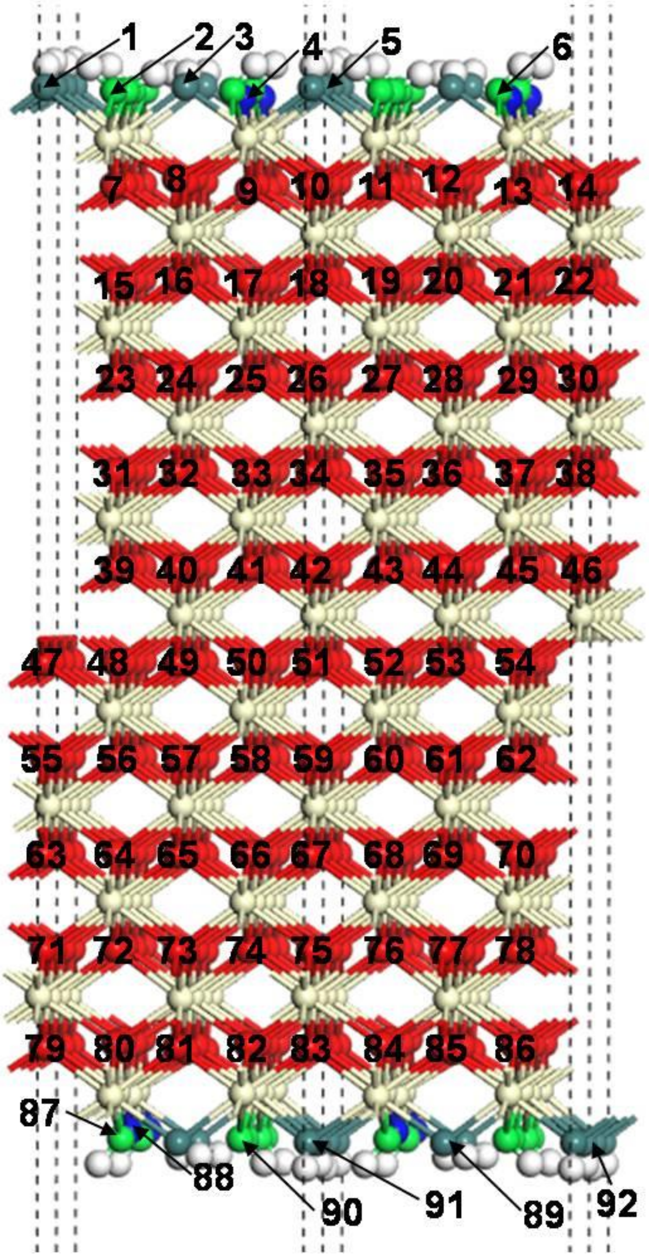

Supplementary Figure 17. Calculated structure of the O-t model with three H_2O molecules dissociatively adsorbed on each surface unit. Oxygen ions with the same chemical environment (thus the same NMR parameters) are labeled with the same number. Red, off-white and white spheres represent internal oxygen, cerium and hydrogen ions, respectively. Surface oxygen groups with different chemical shifts are in different colors.

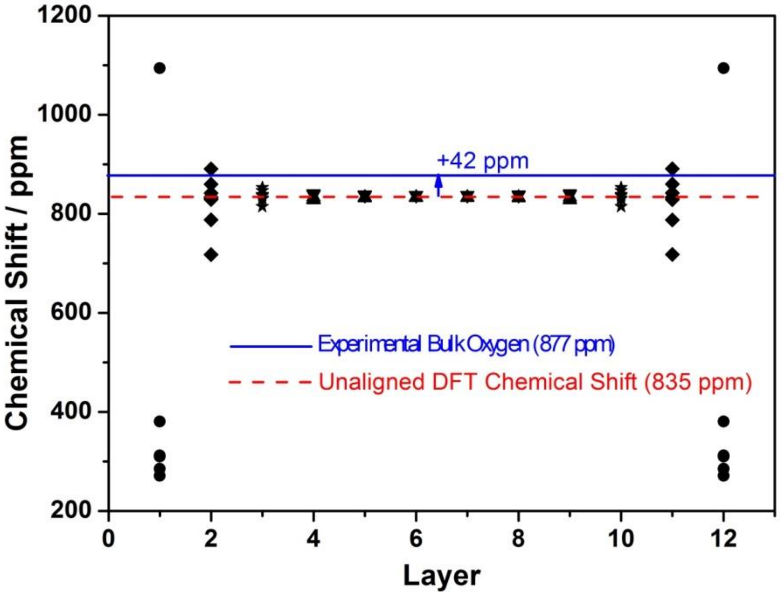

Supplementary Figure 18. The determination of the ^{17}O reference chemical shift (δ_{ref}) from the O-t model with three H_2O molecules dissociatively adsorbed on each surface unit. Spheres, diamonds, stars and triangles represent the unaligned DFT chemical shifts of oxygen ions at the top/bottom 1st, 2nd and 3rd surface layers and the inner part of the structure ("bulk"). The red dashed line shows the average value of the unaligned DFT chemical shifts of the oxygen ions in the "bulk".

Supplementary Table 7. Calculated ^{17}O NMR parameters for oxygen ions in the O-t model with three H_2O molecules dissociatively adsorbed on each surface unit. Isotropic chemical shifts (δ_{iso}), quadrupolar parameters (C_Q and η) and calculated NMR shifts (center of gravity, δ_{CG}) for each resonance are shown. The corresponding structure is presented in Supplementary Figure 17.

O_{ion} No.	δ_{iso} / ppm	C_Q / MHz	η	δ_{CG} / ppm	Assignment
1	327	7.05	0.06	226	Surface -OH
2	353	6.62	0.16	263	Surface -OH
3	423	5.54	0.12	360	Surface -OH
4	1136	0.37	0.92	1136	1st Layer O_{2C}
5	314	7.29	0.17	205	Surface -OH
6	313	6.94	0.21	213	Surface -OH
7	874	0.42	0.53	874	2nd Layer O_{4C}
8	830	0.60	0.66	829	2nd Layer O_{4C}
9	876	0.28	0.18	876	2nd Layer O_{4C}
10	761	0.34	0.21	761	2nd Layer O_{4C}
11	871	0.18	0.48	871	2nd Layer O_{4C}
12	884	0.30	0.97	884	2nd Layer O_{4C}
13	933	0.09	0.12	933	2nd Layer O_{4C}
14	902	0.31	0.48	902	2nd Layer O_{4C}
15	889	0.08	0.36	889	3rd Layer O_{4C}
16	872	0.22	0.99	872	3rd Layer O_{4C}
17	881	0.13	0.73	881	3rd Layer O_{4C}
18	874	0.10	0.57	874	3rd Layer O_{4C}
19	895	0.14	0.52	895	3rd Layer O_{4C}
20	857	0.20	0.77	857	3rd Layer O_{4C}
21	879	0.13	0.82	879	3rd Layer O_{4C}
22	857	0.20	0.71	857	3rd Layer O_{4C}
23	883	0.09	0.86	883	Bulk Like
24	876	0.17	0.53	876	Bulk Like
25	881	0.07	0.24	881	Bulk Like
26	871	0.12	0.55	871	Bulk Like
27	881	0.12	0.33	881	Bulk Like
28	870	0.13	0.80	870	Bulk Like
29	883	0.09	0.77	883	Bulk Like
30	872	0.10	0.47	872	Bulk Like
31	881	0.10	0.38	881	Bulk Like
32	874	0.13	0.42	874	Bulk Like
33	881	0.08	0.72	881	Bulk Like
34	876	0.11	0.28	876	Bulk Like
35	879	0.10	0.29	879	Bulk Like
36	879	0.09	0.46	879	Bulk Like
37	874	0.11	0.25	874	Bulk Like
38	876	0.11	0.20	876	Bulk Like
39	879	0.10	0.13	879	Bulk Like
40	875	0.12	0.19	875	Bulk Like
41	880	0.09	0.21	880	Bulk Like
42	875	0.11	0.18	875	Bulk Like
43	879	0.10	0.09	879	Bulk Like

44	876	0.11	0.14	876	Bulk Like
45	880	0.10	0.19	880	Bulk Like
46	876	0.11	0.12	876	Bulk Like
47	876	0.11	0.02	876	Bulk Like
48	880	0.10	0.10	880	Bulk Like
49	876	0.12	0.02	876	Bulk Like
50	879	0.10	0.03	879	Bulk Like
51	875	0.11	0.05	875	Bulk Like
52	879	0.09	0.12	879	Bulk Like
53	875	0.12	0.07	875	Bulk Like
54	880	0.10	0.06	880	Bulk Like
55	876	0.11	0.19	876	Bulk Like
56	879	0.09	0.33	879	Bulk Like
57	874	0.11	0.21	874	Bulk Like
58	879	0.10	0.18	879	Bulk Like
59	876	0.12	0.23	876	Bulk Like
60	881	0.08	0.64	881	Bulk Like
61	874	0.13	0.38	874	Bulk Like
62	881	0.10	0.26	881	Bulk Like
63	872	0.10	0.41	872	Bulk Like
64	884	0.09	0.75	884	Bulk Like
65	871	0.13	0.74	871	Bulk Like
66	882	0.12	0.32	882	Bulk Like
67	871	0.13	0.48	871	Bulk Like
68	882	0.07	0.30	882	Bulk Like
69	876	0.18	0.49	876	Bulk Like
70	884	0.09	0.92	884	Bulk Like
71	857	0.20	0.74	857	10th Layer O_{4C}
72	880	0.13	0.86	880	10th Layer O_{4C}
73	857	0.20	0.72	857	10th Layer O_{4C}
74	895	0.14	0.55	895	10th Layer O_{4C}
75	874	0.11	0.53	874	10th Layer O_{4C}
76	881	0.14	0.62	881	10th Layer O_{4C}
77	872	0.23	0.96	872	10th Layer O_{4C}
78	889	0.08	0.40	889	10th Layer O_{4C}
79	901	0.30	0.48	901	11th Layer O_{4C}
80	933	0.28	0.91	933	11th Layer O_{4C}
81	884	0.30	0.97	884	11th Layer O_{4C}
82	871	0.18	0.50	871	11th Layer O_{4C}
83	761	0.34	0.21	761	11th Layer O_{4C}
84	876	0.28	0.18	876	11th Layer O_{4C}
85	830	0.61	0.66	830	11th Layer O_{4C}
86	874	0.41	0.52	874	11th Layer O_{4C}
87	312	6.94	0.21	213	Surface -OH
88	1136	0.37	0.92	1136	12th Layer O_{2C}
89	423	5.56	0.13	360	Surface -OH
90	353	6.62	0.16	263	Surface -OH
91	314	7.30	0.17	205	Surface -OH
92	327	7.04	0.06	226	Surface -OH

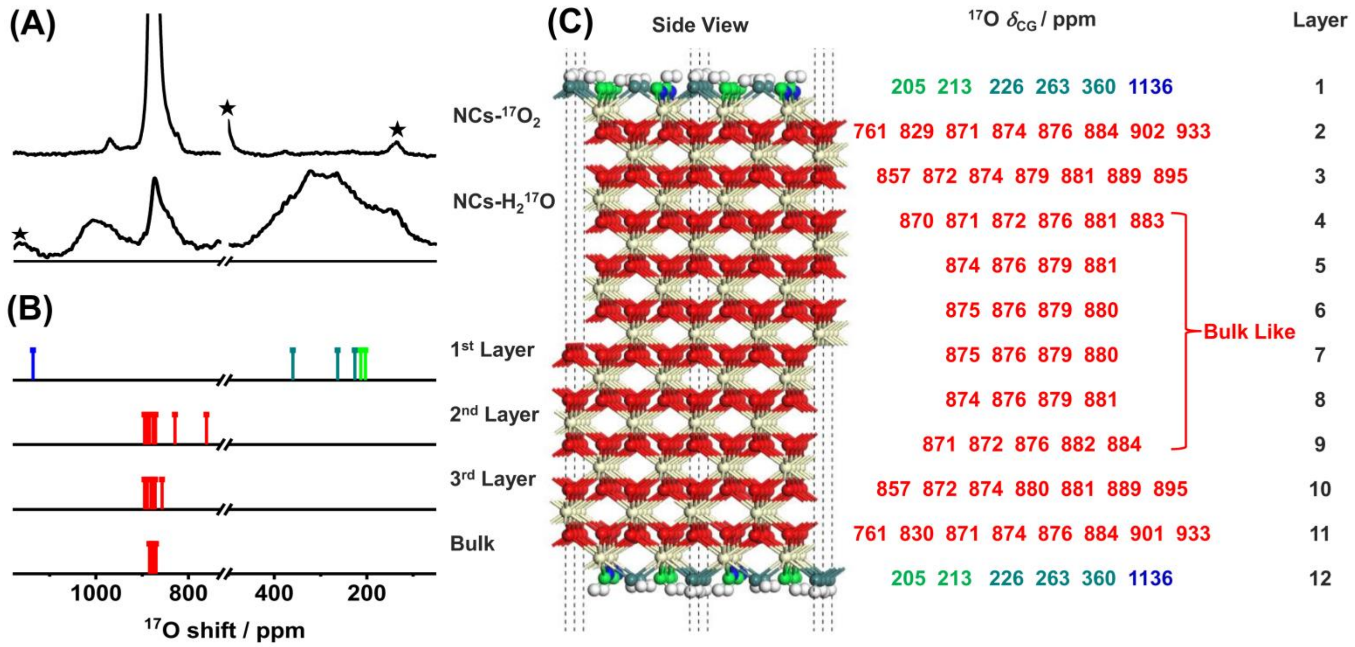

Supplementary Figure 19. ^{17}O NMR spectra, calculated ^{17}O NMR shifts and the O-t structure model with three H_2O molecules dissociatively adsorbed on each surface unit. (A) ^{17}O solid-state NMR spectra of ceria NCs-$^{17}O_2$ and NCs-$H_2{}^{17}O$. (B) The summarized ^{17}O NMR shifts (δ_{CG}s) predicted in the O-t model with three H_2O molecules dissociatively adsorbed on each surface unit shown in (C). (C) Model used in DFT calculations with calculated shifts (δ_{CG}s) for oxygen ions (labeled in Supplementary Figure 17) and the layer number shown on the right. The calculated ^{17}O NMR parameters for each oxygen ion are shown in Supplementary Table 7.

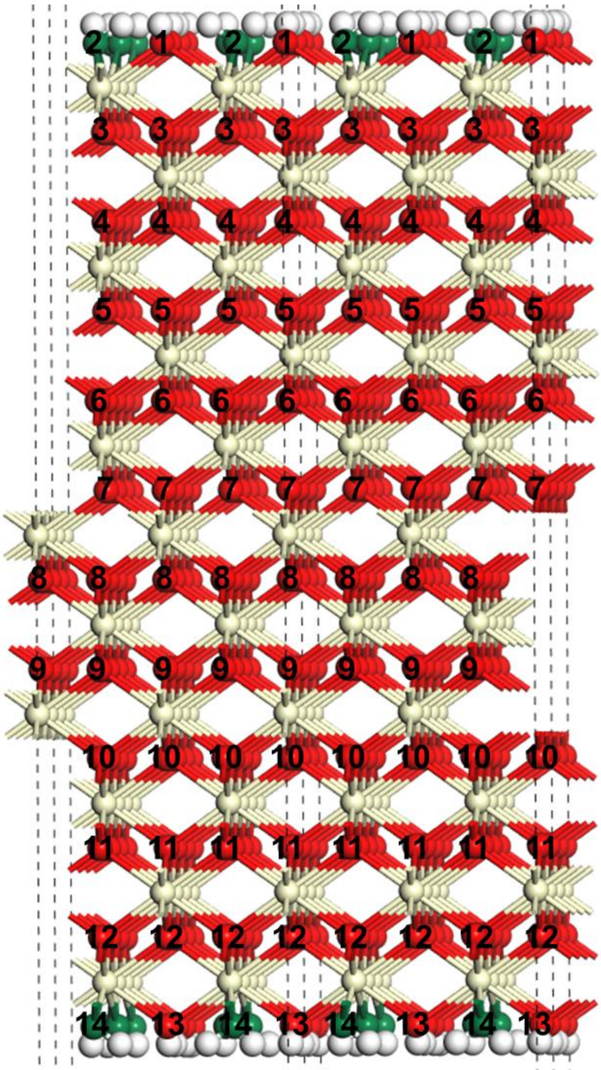

Supplementary Figure 20. Calculated structure of the O-t model with four H_2O molecules dissociatively adsorbed on each surface unit (monolayer hydroxylated). Oxygen ions with the same chemical environment (thus the same NMR parameters) are labeled with the same number. Red, off-white and white spheres represent internal oxygen, cerium and hydrogen ions, respectively. Surface oxygen groups with different chemical shifts are in different colors.

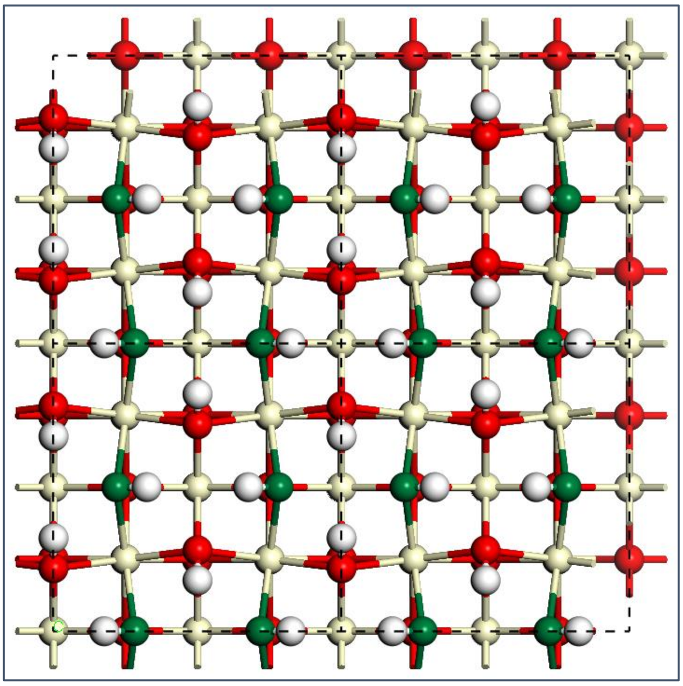

Supplementary Figure 21. Top view of the calculated structure of the O-t model with four H_2O molecules dissociatively adsorbed on each surface unit (monolayer hydroxylated). Red, off-white and white spheres represent internal oxygen, cerium and hydrogen ions, respectively. It can be seen that eight bridging hydroxyls (-OH_B) form from the four dissociated H_2O molecules in one O-t surface unit.

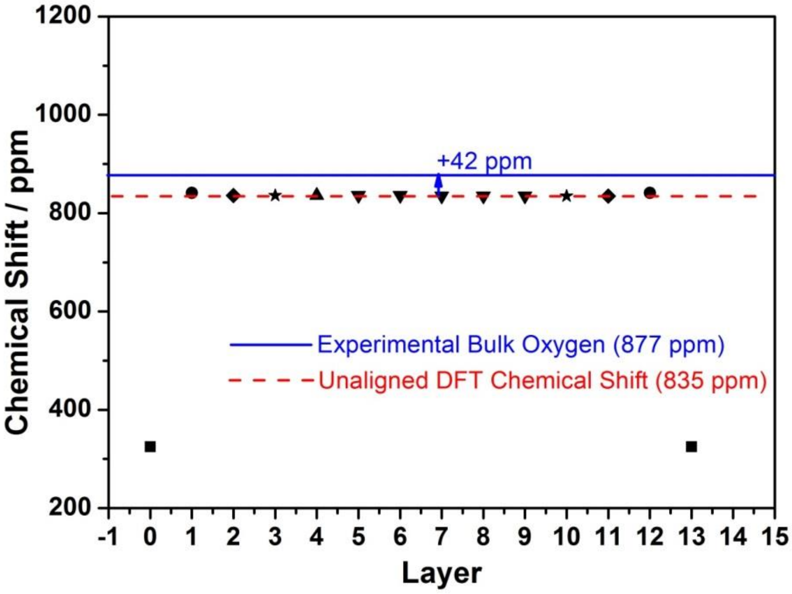

Supplementary Figure 22. The determination of the ^{17}O reference chemical shift (δ_{ref}) from the O-t model with four H_2O molecules dissociatively adsorbed on each surface unit (monolayer hydroxylated). Spheres, diamonds, stars and triangles represent the unaligned DFT chemical shifts of oxygen ions at the top/bottom 1st, 2nd and 3rd surface layers and the inner part of the structure ("bulk"). The red dashed line shows the average value of the unaligned DFT chemical shifts of the oxygen ions in the "bulk".

Supplementary Table 8. Calculated ^{17}O NMR parameters for oxygen ions in the O-t model with four H_2O molecules dissociatively adsorbed on each surface unit (monolayer hydroxylated). Isotropic chemical shifts (δ_{iso}), quadrupolar parameters (C_Q and η) and calculated NMR shifts (center of gravity, δ_{CG}) for each resonance are shown. The corresponding structure is presented in Supplementary Figure 20.

O_{ion} No.	δ_{iso} / ppm	C_Q / MHz	η	δ_{CG} / ppm	Assignment
1	367	6.14	0.12	290	Surface -OH
2	367	6.16	0.13	289	Surface -OH
3	883	0.11	0.75	883	2nd Layer O_{4C}
4	877	0.11	0.08	877	3rd Layer O_{4C}
5	877	0.12	0.03	877	Bulk Like
6	877	0.12	0.02	877	Bulk Like
7	877	0.11	0.02	877	Bulk Like
8	877	0.11	0.14	877	Bulk Like
9	877	0.10	0.12	877	Bulk Like
10	877	0.10	0.03	877	Bulk Like
11	877	0.11	0.04	877	10th Layer O_{4C}
12	883	0.11	0.86	883	11th Layer O_{4C}
13	367	6.15	0.13	289	Surface -OH
14	367	6.15	0.12	290	Surface -OH

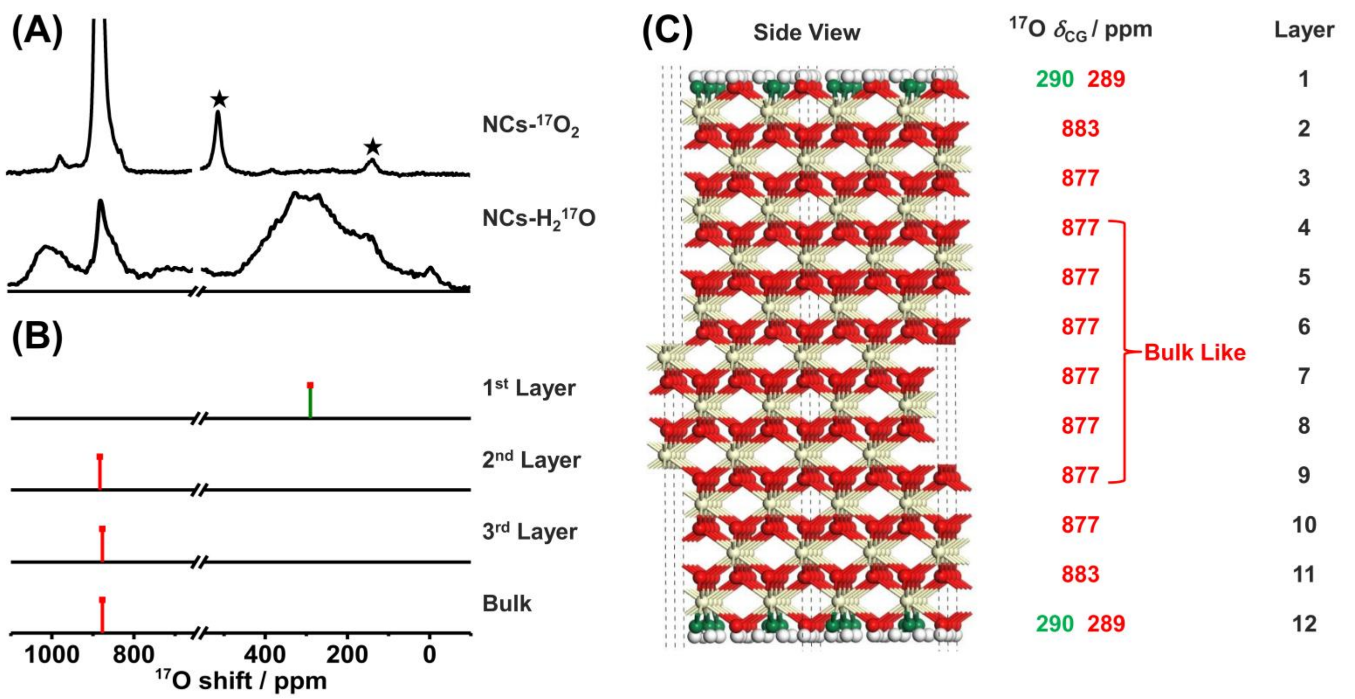

Supplementary Figure 23. ^{17}O NMR spectra, calculated ^{17}O NMR shifts and the O-t structure model with four H_2O molecules dissociatively adsorbed on each surface unit. (A) ^{17}O solid-state NMR spectra of ceria NCs-$^{17}O_2$ and NCs-$H_2{}^{17}O$. (B) The summarized ^{17}O NMR shifts (δ_{CG}s) predicted in the O-t model with four H_2O molecules dissociatively adsorbed on each surface unit (monolayer hydroxylated) shown in (C). (C) Model used in DFT calculations with calculated shifts (δ_{CG}s) for oxygen ions (labeled in Supplementary Figure 20) and the layer number shown on the right. The calculated ^{17}O NMR parameters for each oxygen ion are shown in Supplementary Table 8.

Section D: Calculations of the adsorption site and energies of H_2O on CeO_4-t and O-t surface units

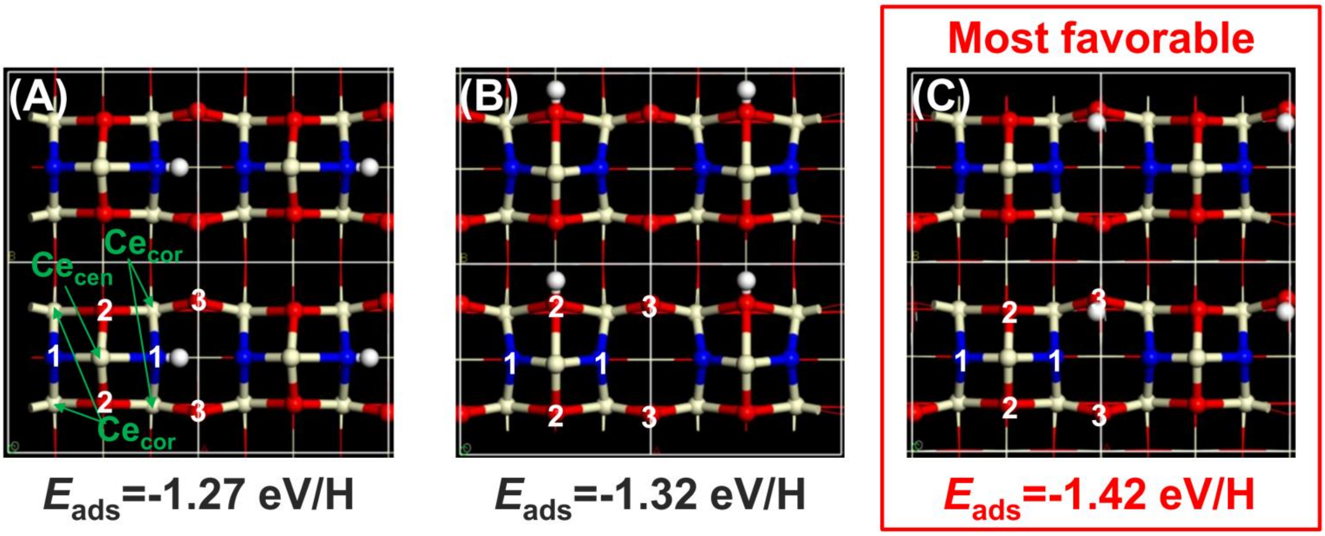

Supplementary Figure 24. Models of four CeO_4-t surface units with one hydrogen ion adsorbed on each surface unit. There is one cerium ion at the center (Ce_{cen}), which is on the top of the surface, and four cerium ions at the corners (Ce_{cor}). Cerium ions are in off-white and hydrogen ions are in white, while oxygen ions are labeled with blue or red and are indexed with numbers according to their positions (O1, O2 and O3). Hydrogen ions are bound to O1, O2 and O3 in (A), (B) and (C), respectively. The adsorption energies for a single hydrogen ion in one unit in (A), (B) and (C) are -1.27, -1.32 and -1.42 eV, respectively, suggesting that the most favorable adsorption site for one hydrogen ion is O3.

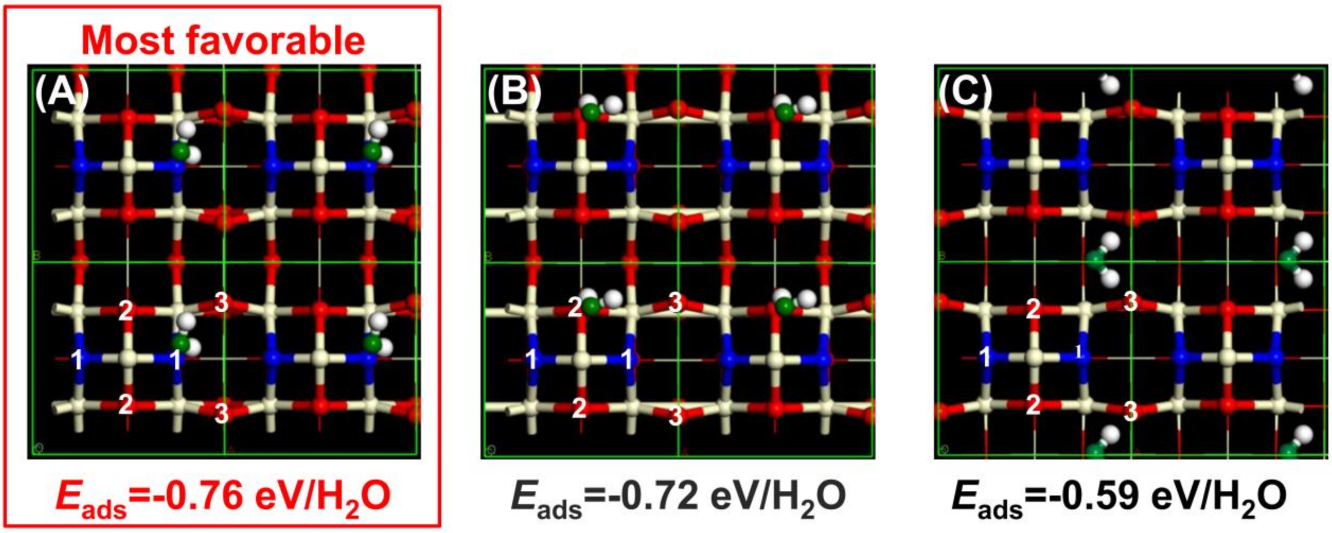

Supplementary Figure 25. Models of four CeO_4-t surface units with one H_2O molecule molecularly adsorbed on each surface unit. All the ions are labeled the same as in Supplementary Figure 24, except that the oxygen ions of the adsorbed H_2O molecules are in green. H_2O molecules are bound in HOH-O1, HOH-O2 and O3-HOH-O3 configurations, in (A), (B) and (C), respectively. The adsorption energies for a single H_2O molecule in one unit in (A), (B) and (C) are -0.76, -0.72 and -0.59 eV, respectively, suggesting that the most favorable adsorption site is O1 for the model with one molecularly adsorbed H_2O molecule per unit.

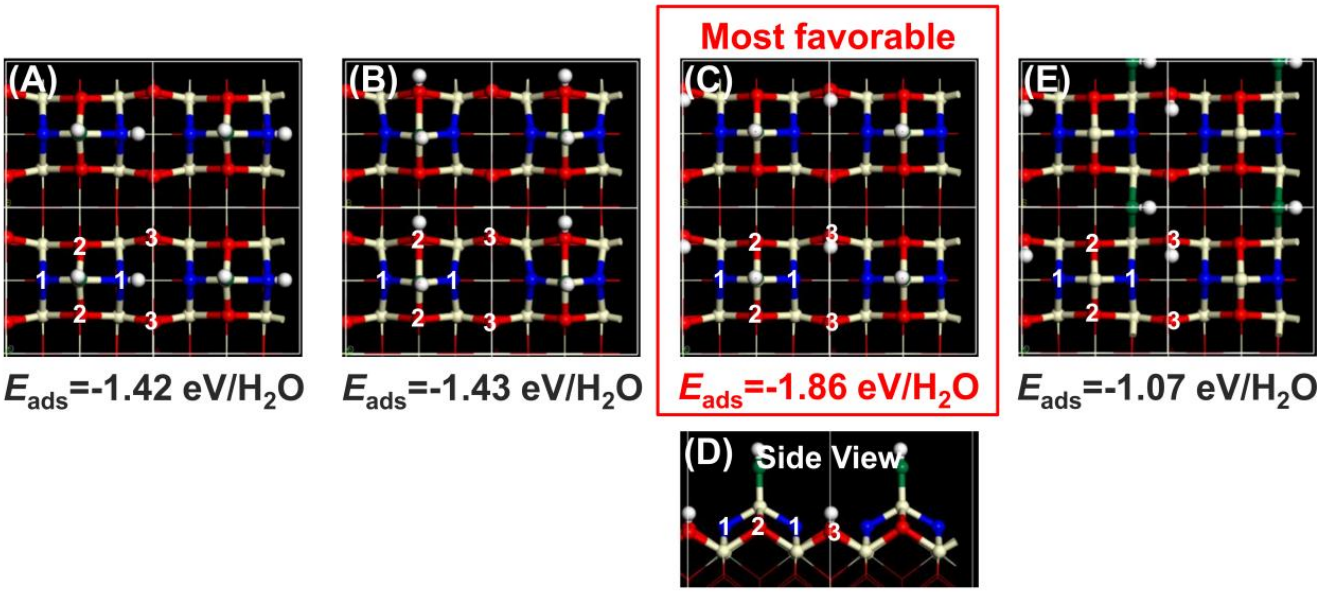

Supplementary Figure 26. Models of four CeO_4-t surface units with one H_2O molecule dissociatively adsorbed on each surface unit. All the ions are labeled the same as in Supplementary Figure 25. The dissociation of one H_2O molecule per unit generates one hydrogen ion and one hydroxyl group. The hydrogen ions are adsorbed on O1 (A), O2 (B), and O3 (C and E), and the hydroxyl groups are bound to Ce_{cen} in (A – C) and between two Ce_{cor} in (E). (D) is the side view of (C). Hydroxyl groups bound to Ce_{cen} ion are terminal hydroxyl sites (-OH_T) while the rest of the hydroxyl groups are bridging hydroxyl sites (-OH_B). The dissociative adsorption energies per surface unit are approximately twice the values for molecular adsorption (Supplementary Figure 24). Therefore, H_2O molecules prefer dissociative adsorption on ceria (100) and only dissociative adsorption models are considered at higher H_2O concentrations. The model in (C) is energetically the most favorable (abbreviated as M_1).

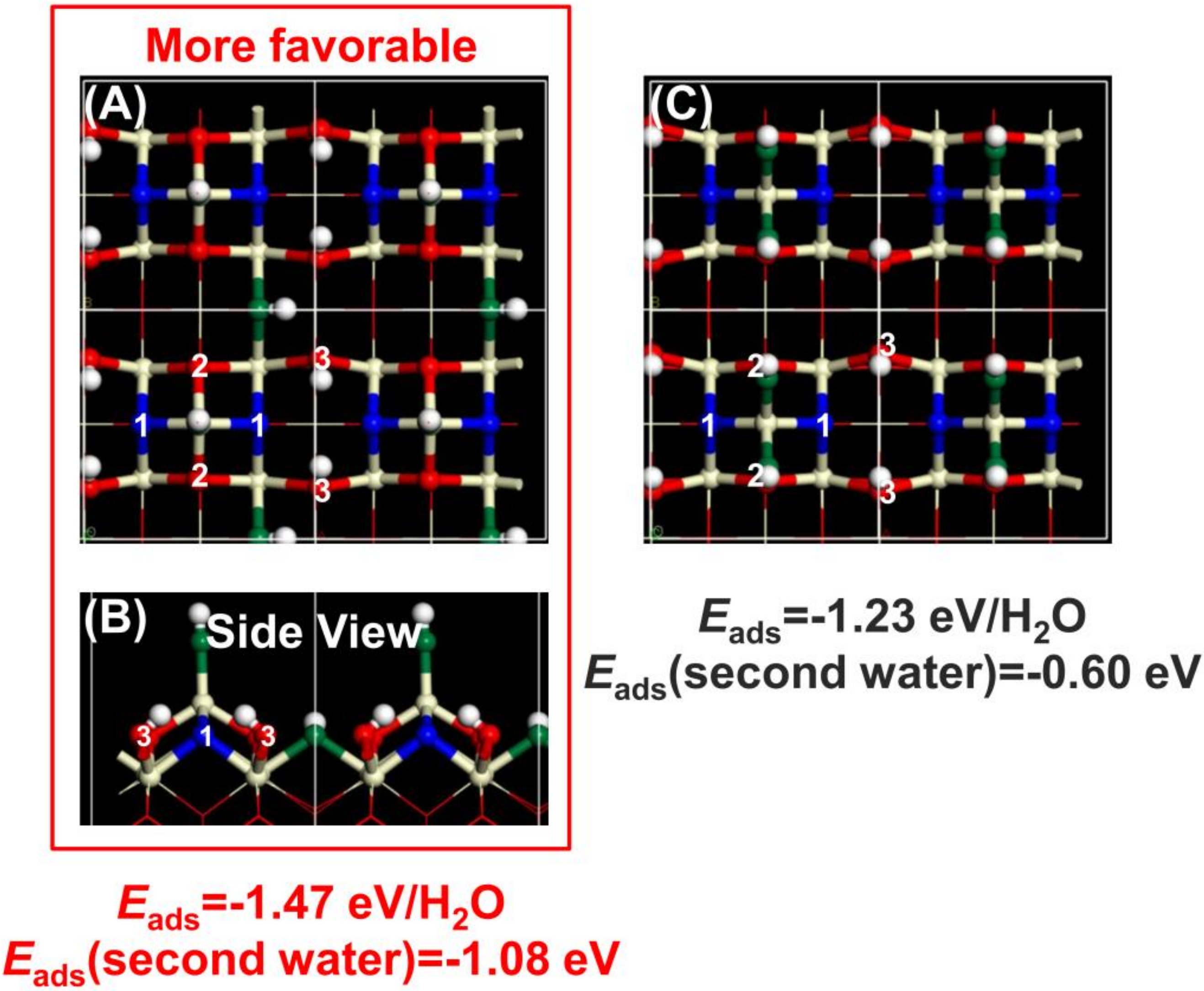

Supplementary Figure 27. Models of four CeO_4-t surface units with two H_2O molecules dissociatively adsorbed on each surface unit. All the ions are labeled the same as Supplementary Figure 25. These models are based on M_1 with a second dissociatively adsorbed H_2O molecule added per unit. The dissociation of a second H_2O molecule per unit generates another hydrogen ion and another hydroxyl group. For each CeO_4-t unit, the generated hydrogen ion and hydroxyl group are bound to another O3 and between two Ce_{cor}, respectively, in (A), while the hydrogen ion and hydroxyl group are bound to another O3 and Ce_{cen} in (C). (B) is the side view of (A). The adsorption energies of the second H_2O molecule per unit of (A) and (C) are -1.08 and -0.60 eV, respectively, indicating that the model in (A) (abbreviated as M_2) is energetically more favorable. The average dissociative adsorption energy per H_2O molecule per surface unit of M_2 is -1.47 eV.

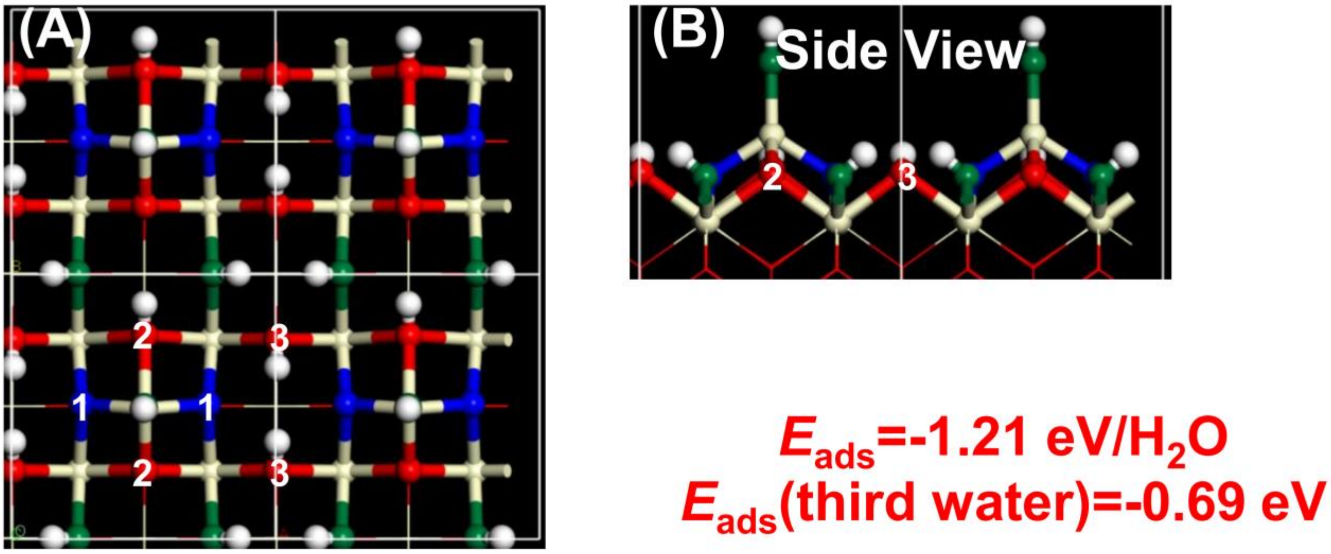

Supplementary Figure 28. The model of four CeO_4-t surface units with three H_2O molecules dissociatively adsorbed on each surface unit. All the ions are labeled and colored the same as in Supplementary Figure 25. This model is based on M_2 with a third dissociatively adsorbed H_2O molecule added, thus adding one hydrogen ion and hydroxyl group per surface unit. For each CeO_4-t surface unit, the generated hydrogen ion and hydroxyl group are bound to O2 and between two Ce_{cor}, respectively (A). (B) is the side view of (A). This model is abbreviated as M_3. The dissociative adsorption energy of the third H_2O molecule per surface unit is calculated as -0.69 eV which is approximately that of the molecularly adsorbed H_2O, as shown in Supplementary Figure 25. This suggests that if more H_2O molecules were added to M_3, they would be more likely to adsorb molecularly. The average dissociative adsorption per H_2O molecule per unit of M_3 is -1.21 eV.

Supplementary Table 9. Calculated average adsorption energies of each water molecule (E_{ads}, in eV / H_2O) on the (100) O-t or CeO_4-t surfaces at molecular adsorption or dissociative adsorption (note that only the structure with the most negative energy is considered for each case), in comparison to the values reported previously.

Number of Water Molecules per Surface Unit		O-t Surface Unit		CeO_4-t Surface Unit	
		Molecular / eV	Dissociative / eV	Molecular / eV	Dissociative / eV
1	Our work	–	-1.47	-0.76	-1.86
	Ref 8.	-0.94[a]	-1.65[a]	–	–
2	Our work	–	-1.21	–	-1.47
	Ref 8.	–	-1.53[a]	–	–
3	Our work	–	-1.38	–	-1.21
4	Our work	–	-1.31	–	–

[a] PBE + *U* (4.5 eV for Ce 4*f*) + D2 value reported for a *p*(2 × 2) surface cell;[8]

Section E: DFT calculations of ^{17}O NMR parameters on CeO_4-t surfaces with different numbers of dissociated water molecules

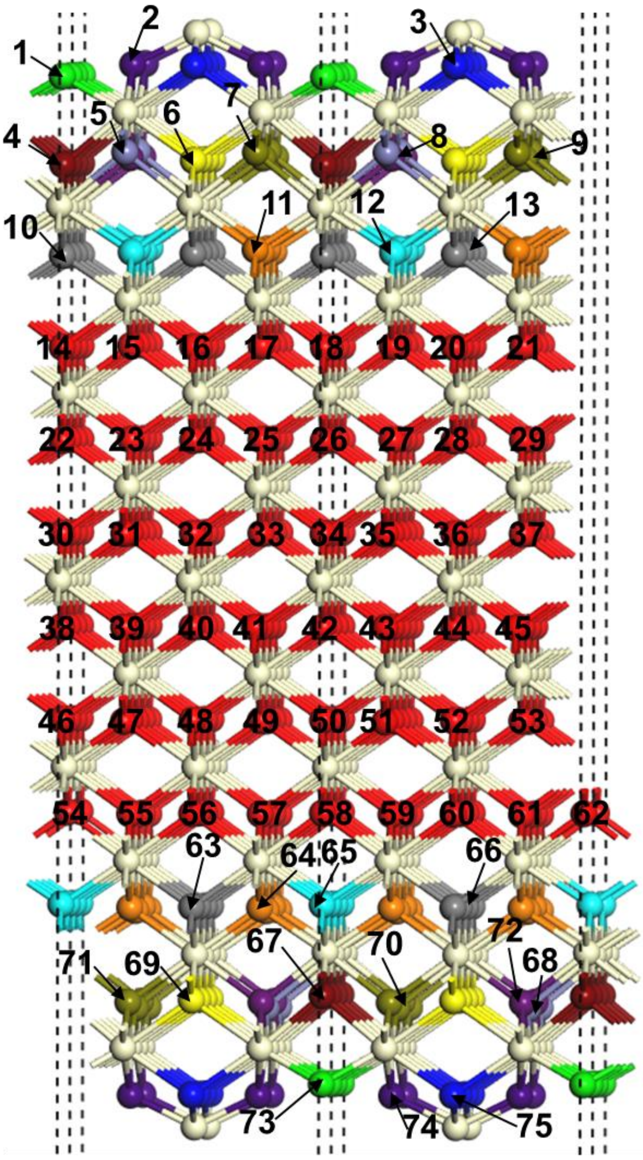

Supplementary Figure 29. Calculated structure of the clean CeO_4-t model (M_0). Oxygen ions with the same chemical environment (thus the same NMR parameters) are labeled with the same number (Supplementary Table 10). Red and off-white spheres represent bulk oxygen and cerium ions, respectively. Surface oxygen groups with different chemical shifts are in different colors.

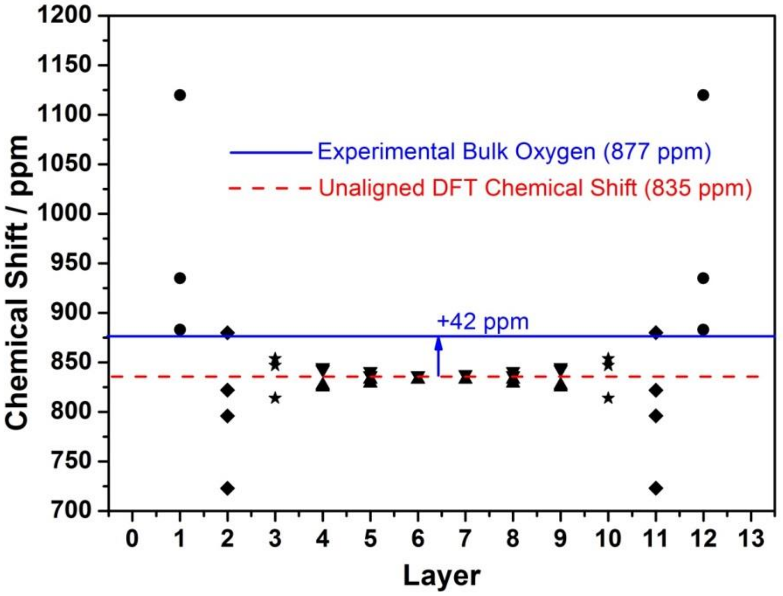

Supplementary Figure 30. The determination of the ^{17}O reference chemical shift (δ_{ref}) for M_0. Spheres, diamonds, stars and triangles represent the unaligned DFT chemical shifts of oxygen ions at the top/bottom 1st, 2nd and 3rd surface layers and the inner part of the structure ("bulk"). The red dashed line shows the average value of the unaligned DFT chemical shifts of the oxygen ions in the "bulk".

Supplementary Table 10. Calculated ^{17}O NMR parameters (isotropic chemical shifts (δ_{iso}), quadrupolar parameters (C_Q and η) and calculated NMR shifts (center of gravity, δ_{CG}s)) for oxygen ions in M_0. The corresponding structure is presented in Supplementary Figure 29.

O_{ion} No.	δ_{iso} / ppm	C_Q / MHz	η	δ_{CG} / ppm	Assignment
1	1162	0.72	0.81	1161	1st Layer O_{2C}
2	977	0.52	0.36	976	1st Layer O_{3C}
3	925	0.71	0.63	924	1st Layer O_{3C}
4	864	0.55	0.12	863	2nd Layer O_{4C}
5	838	0.73	0.51	837	2nd Layer O_{4C}
6	922	0.07	0.99	922	2nd Layer O_{4C}
7	838	0.73	0.51	837	2nd Layer O_{4C}
8	765	0.43	0.44	765	2nd Layer O_{4C}
9	765	0.43	0.44	765	2nd Layer O_{4C}
10	889	0.21	0.01	889	3rd Layer O_{4C}
11	856	0.32	1.00	856	3rd Layer O_{4C}
12	856	0.18	0.32	856	3rd Layer O_{4C}
13	896	0.19	0.47	896	3rd Layer O_{4C}
14	887	0.10	0.66	887	Bulk Like
15	867	0.11	0.73	867	Bulk Like
16	885	0.71	0.80	884	Bulk Like
17	869	0.20	0.81	869	Bulk Like
18	887	0.10	0.66	887	Bulk Like
19	867	0.11	0.73	867	Bulk Like
20	885	0.71	0.80	884	Bulk Like
21	869	0.20	0.81	869	Bulk Like
22	883	0.07	0.83	883	Bulk Like
23	875	0.11	0.26	875	Bulk Like
24	880	0.08	0.46	880	Bulk Like
25	871	0.13	0.40	871	Bulk Like
26	883	0.07	0.83	883	Bulk Like
27	875	0.11	0.26	875	Bulk Like
28	880	0.08	0.46	880	Bulk Like
29	871	0.13	0.40	871	Bulk Like
30	879	0.08	0.15	879	Bulk Like
31	875	0.11	0.09	875	Bulk Like
32	879	0.09	0.13	879	Bulk Like
33	875	0.11	0.09	875	Bulk Like
34	879	0.08	0.15	879	Bulk Like
35	875	0.11	0.09	875	Bulk Like
36	879	0.09	0.13	879	Bulk Like
37	875	0.11	0.09	875	Bulk Like
38	879	0.08	0.24	879	Bulk Like
39	875	0.10	0.06	875	Bulk Like
40	879	0.09	0.17	879	Bulk Like
41	875	0.12	0.20	875	Bulk Like
42	879	0.08	0.24	879	Bulk Like
43	875	0.10	0.06	875	Bulk Like
44	879	0.09	0.17	879	Bulk Like
45	875	0.12	0.20	875	Bulk Like

46	883	0.07	1.00	883	Bulk Like
47	875	0.11	0.28	875	Bulk Like
48	879	0.09	0.50	879	Bulk Like
49	871	0.13	0.62	871	Bulk Like
50	883	0.07	1.00	883	Bulk Like
51	875	0.11	0.28	875	Bulk Like
52	879	0.09	0.50	879	Bulk Like
53	871	0.13	0.62	871	Bulk Like
54	886	0.10	0.64	886	Bulk Like
55	866	0.11	0.58	866	Bulk Like
56	885	0.11	0.76	885	Bulk Like
57	869	0.18	0.93	869	Bulk Like
58	886	0.10	0.64	886	Bulk Like
59	866	0.11	0.58	866	Bulk Like
60	885	0.11	0.76	885	Bulk Like
61	869	0.18	0.93	869	Bulk Like
62	886	0.10	0.64	886	Bulk Like
63	896	0.18	0.43	896	10th Layer O_{4C}
64	856	0.32	0.94	856	10th Layer O_{4C}
65	856	0.21	0.06	856	10th Layer O_{4C}
66	889	0.19	0.43	889	10th Layer O_{4C}
67	864	0.55	0.14	863	11th Layer O_{4C}
68	838	0.72	0.52	837	11th Layer O_{4C}
69	922	0.36	0.92	922	11th Layer O_{4C}
70	838	0.72	0.52	837	11th Layer O_{4C}
71	765	0.44	0.43	765	11th Layer O_{4C}
72	765	0.44	0.43	765	11th Layer O_{4C}
73	1162	0.72	0.80	1161	12th Layer O_{2C}
74	977	0.52	0.34	976	12th Layer O_{3C}
75	925	0.70	0.64	924	12th Layer O_{3C}

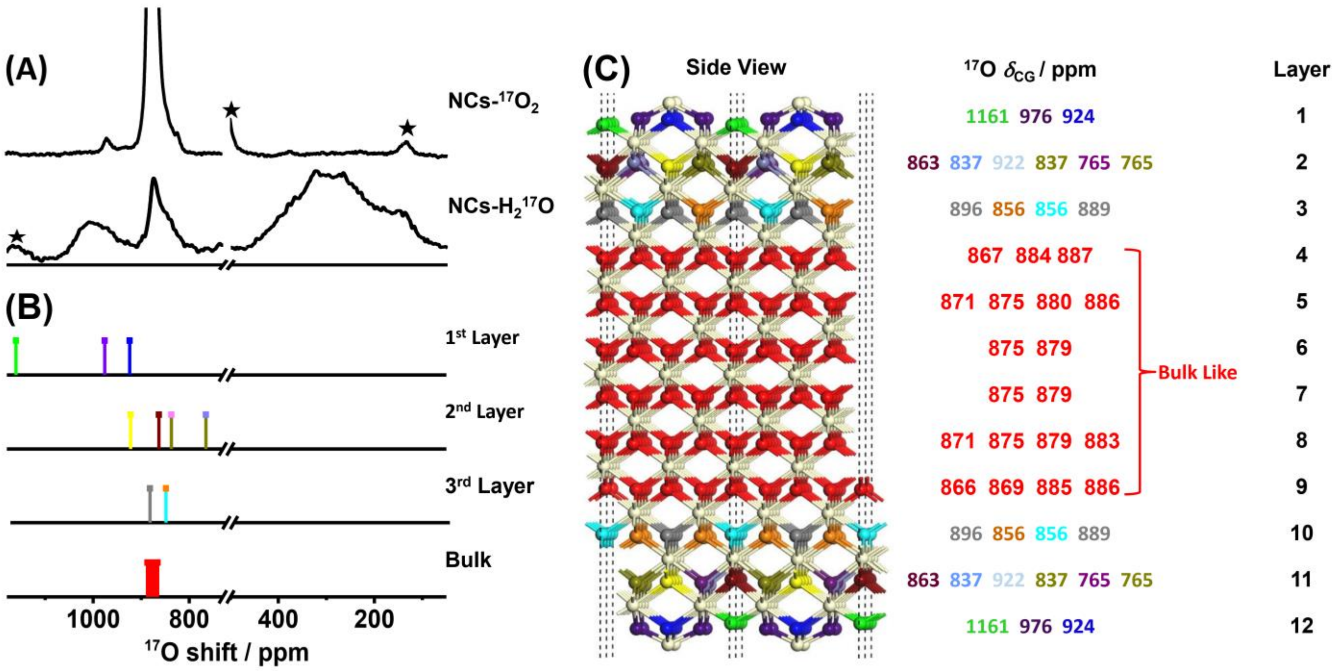

Supplementary Figure 31. ^{17}O NMR spectrum, calculated ^{17}O NMR shifts and the structure model of M_0. (A) ^{17}O solid-state NMR spectra of ceria NCs-$^{17}O_2$ and NCs-$H_2{}^{17}O$. (B) The summarized ^{17}O NMR shifts (δ_{CG}s) of oxygen ions in M_0 shown in (C). (C) The CeO_4-t model used in DFT calculations with calculated shifts (δ_{CG}s) for oxygen ions (labeled in Supplementary Figure 29) and the layer number shown on the right. The calculated ^{17}O NMR parameters for each oxygen ion are shown in Supplementary Table 10.

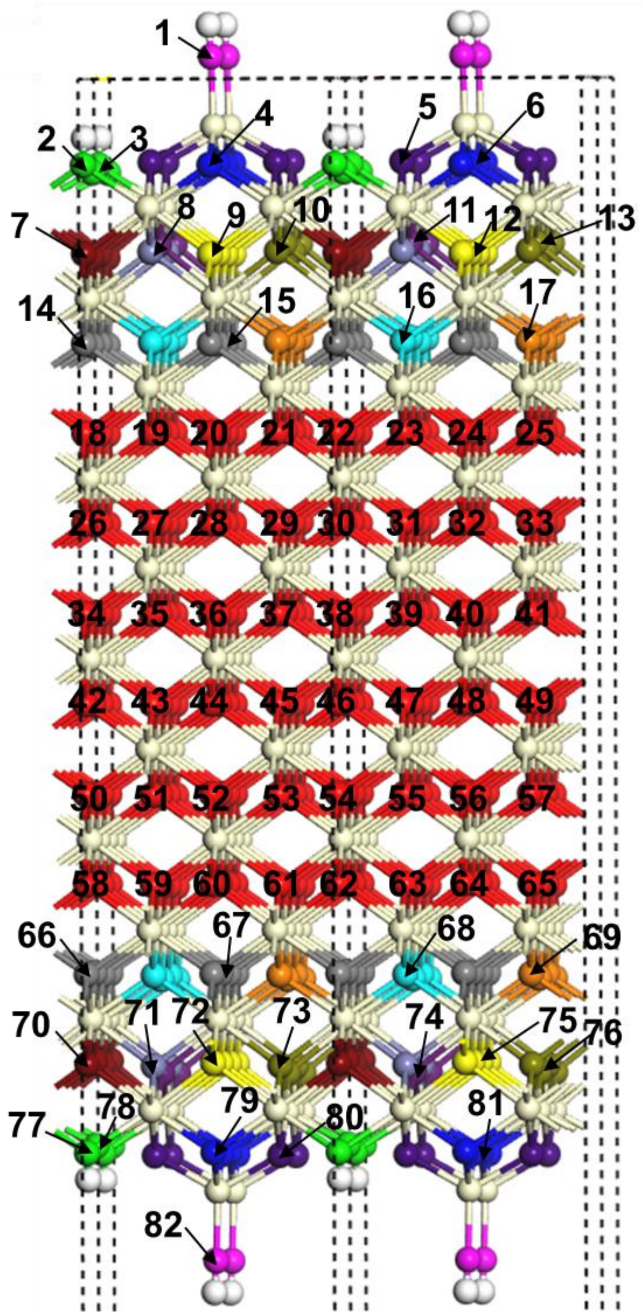

Supplementary Figure 32. Calculated structure of the hydrated CeO_4-t model with one H_2O molecule dissociatively adsorbed on each surface unit (M_1). Oxygen ions with the same chemical environment (thus the same NMR parameters) are labeled with the same number (Supplementary Table 11). Red, off-white and white spheres represent bulk oxygen, cerium and hydrogen ions, respectively. Surface oxygen groups with different chemical shifts are in different colors.

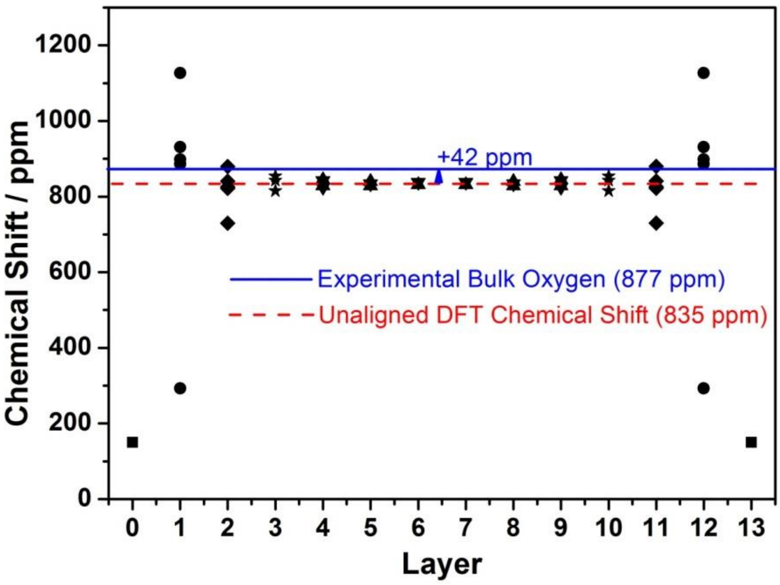

Supplementary Figure 33. The determination of the ^{17}O reference chemical shift (δ_{ref}) for M_1. Spheres, diamonds, stars and triangles (overlap from layer 4 to 9 because of the small numerical difference) represent the unaligned DFT chemical shifts of oxygen ions at the top/bottom 1st, 2nd and 3rd surface layers and the inner part of the structure ("bulk"). The red dashed line shows the average value of the unaligned DFT chemical shifts of the oxygen ions in the "bulk".

Supplementary Table 11. Calculated ^{17}O NMR parameters (isotropic chemical shifts (δ_{iso}), quadrupolar parameters (C_Q and η) and calculated NMR shifts (center of gravity, δ_{CG}s)) for oxygen ions in M_1. The corresponding structure is presented in Supplementary Figure 32.

O_{ion} No.	δ_{iso} / ppm	C_Q / MHz	η	δ_{CG} / ppm	Assignment
1	192	7.50	0.01	77	Surface -OH
2	335	6.93	0.20	236	Surface -OH
3	1169	0.64	0.79	1168	1st Layer O_{2C}
4	940	0.43	0.80	940	1st Layer O_{3C}
5	973	0.31	0.55	973	1st Layer O_{3C}
6	928	0.58	0.18	927	1st Layer O_{3C}
7	883	0.43	0.24	883	2nd Layer O_{4C}
8	772	0.31	0.93	772	2nd Layer O_{4C}
9	868	0.32	0.92	868	2nd Layer O_{4C}
10	772	0.32	0.92	772	2nd Layer O_{4C}
11	864	0.65	0.51	863	2nd Layer O_{4C}
12	922	0.38	0.67	922	2nd Layer O_{4C}
13	864	0.66	0.51	863	2nd Layer O_{4C}
14	896	0.13	0.87	896	3rd Layer O_{4C}
15	885	0.19	0.55	885	3rd Layer O_{4C}
16	857	0.34	0.94	857	3rd Layer O_{4C}
17	857	0.34	0.94	857	3rd Layer O_{4C}
18	887	0.11	0.57	887	Bulk Like
19	871	0.21	0.81	871	Bulk Like
20	884	0.01	0.05	884	Bulk Like
21	867	0.12	0.65	867	Bulk Like
22	887	0.11	0.57	887	Bulk Like
23	871	0.21	0.81	871	Bulk Like
24	884	0.01	0.05	884	Bulk Like
25	867	0.12	0.65	867	Bulk Like
26	882	0.10	0.88	882	Bulk Like
27	871	0.15	0.47	871	Bulk Like
28	882	0.10	0.64	882	Bulk Like
29	876	0.12	0.27	876	Bulk Like
30	882	0.10	0.65	882	Bulk Like
31	871	0.15	0.47	871	Bulk Like
32	882	0.10	0.64	882	Bulk Like
33	876	0.12	0.27	876	Bulk Like
34	880	0.10	0.17	880	Bulk Like
35	874	0.13	0.14	874	Bulk Like
36	880	0.10	0.18	880	Bulk Like
37	874	0.13	0.14	874	Bulk Like
38	880	0.10	0.17	880	Bulk Like
39	874	0.13	0.14	874	Bulk Like
40	880	0.10	0.18	880	Bulk Like
41	874	0.13	0.14	874	Bulk Like
42	880	0.10	0.18	880	Bulk Like
43	874	0.14	0.15	874	Bulk Like
44	880	0.10	0.22	880	Bulk Like
45	874	0.14	0.15	874	Bulk Like

46	880	0.10	0.18	880	Bulk Like
47	874	0.14	0.15	874	Bulk Like
48	880	0.10	0.22	880	Bulk Like
49	874	0.14	0.15	874	Bulk Like
50	882	0.10	0.96	882	Bulk Like
51	871	0.15	0.55	871	Bulk Like
52	882	0.10	0.69	882	Bulk Like
53	876	0.12	0.35	876	Bulk Like
54	882	0.10	0.96	882	Bulk Like
55	871	0.15	0.55	871	Bulk Like
56	882	0.10	0.69	882	Bulk Like
57	876	0.12	0.35	876	Bulk Like
58	887	0.12	0.46	887	Bulk Like
59	871	0.21	0.88	871	Bulk Like
60	884	0.10	0.16	884	Bulk Like
61	867	0.10	0.72	867	Bulk Like
62	887	0.12	0.46	887	Bulk Like
63	871	0.21	0.88	871	Bulk Like
64	884	0.10	0.16	884	Bulk Like
65	867	0.10	0.72	867	Bulk Like
66	896	0.13	0.90	896	10th Layer O_{4C}
67	885	0.20	0.54	885	10th Layer O_{4C}
68	857	0.33	0.99	857	10th Layer O_{4C}
69	857	0.33	0.99	857	10th Layer O_{4C}
70	883	0.57	0.03	882	11th Layer O_{4C}
71	772	0.31	0.95	772	11th Layer O_{4C}
72	868	0.30	0.19	868	11th Layer O_{4C}
73	772	0.31	0.92	772	11th Layer O_{4C}
74	864	0.65	0.52	863	11th Layer O_{4C}
75	922	0.38	0.69	922	11th Layer O_{4C}
76	864	0.65	0.52	863	11th Layer O_{4C}
77	335	6.91	0.20	236	Surface -OH
78	1169	0.64	0.78	1168	12th Layer O_{2C}
79	940	0.43	0.80	940	12th Layer O_{3C}
80	973	0.31	0.51	973	12th Layer O_{3C}
81	928	0.58	0.17	927	12th Layer O_{3C}
82	192	7.50	0.01	77	Surface -OH

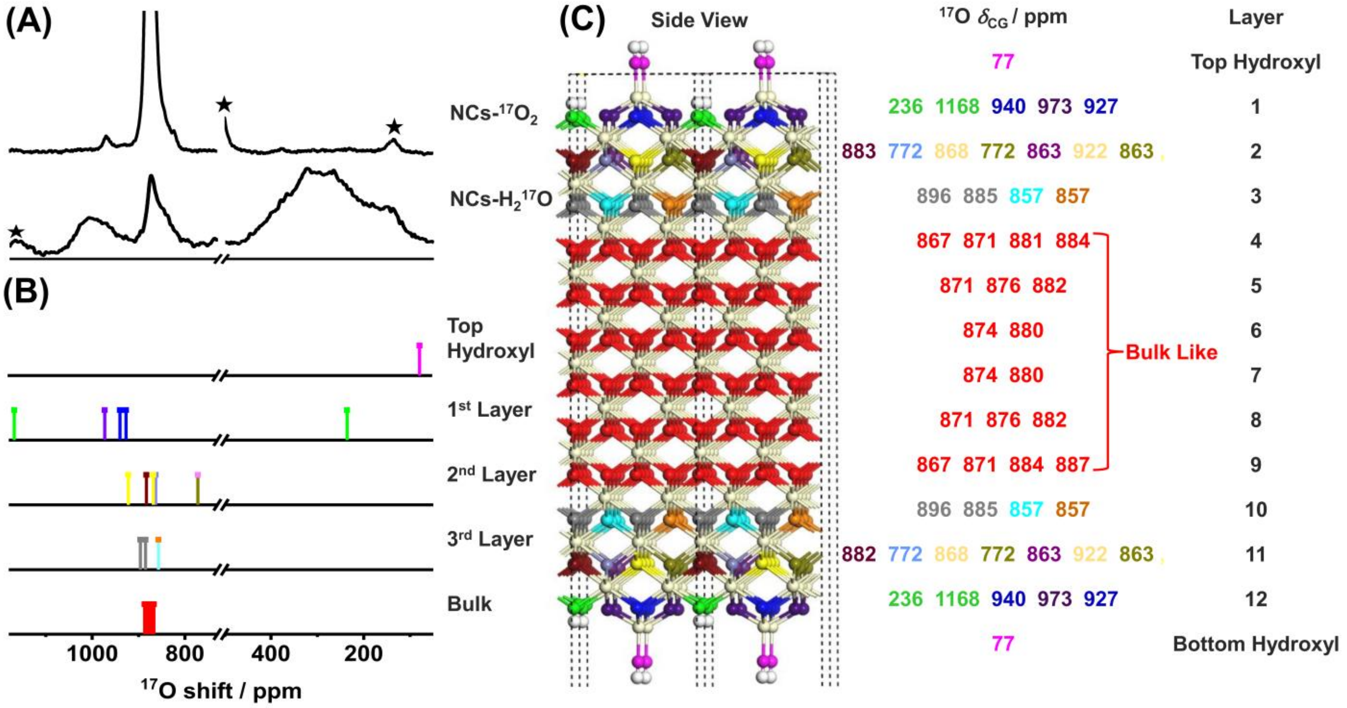

Supplementary Figure 34. ^{17}O NMR spectrum, calculated ^{17}O NMR shifts and the structure model of M_1. (A) ^{17}O solid-state NMR spectra of ceria NCs-$^{17}O_2$ and NCs-$H_2{}^{17}O$. (B) The summarized ^{17}O NMR shifts (δ_{CG}s) of oxygen ions in M_1 shown in (C). (C) The hydrated CeO_4-t model used in DFT calculations with calculated shifts (δ_{CG}s) for oxygen ions (labeled in Supplementary Figure 32) and the layer number shown on the right. The calculated ^{17}O NMR parameters for each oxygen ion are shown in Supplementary Table 11.

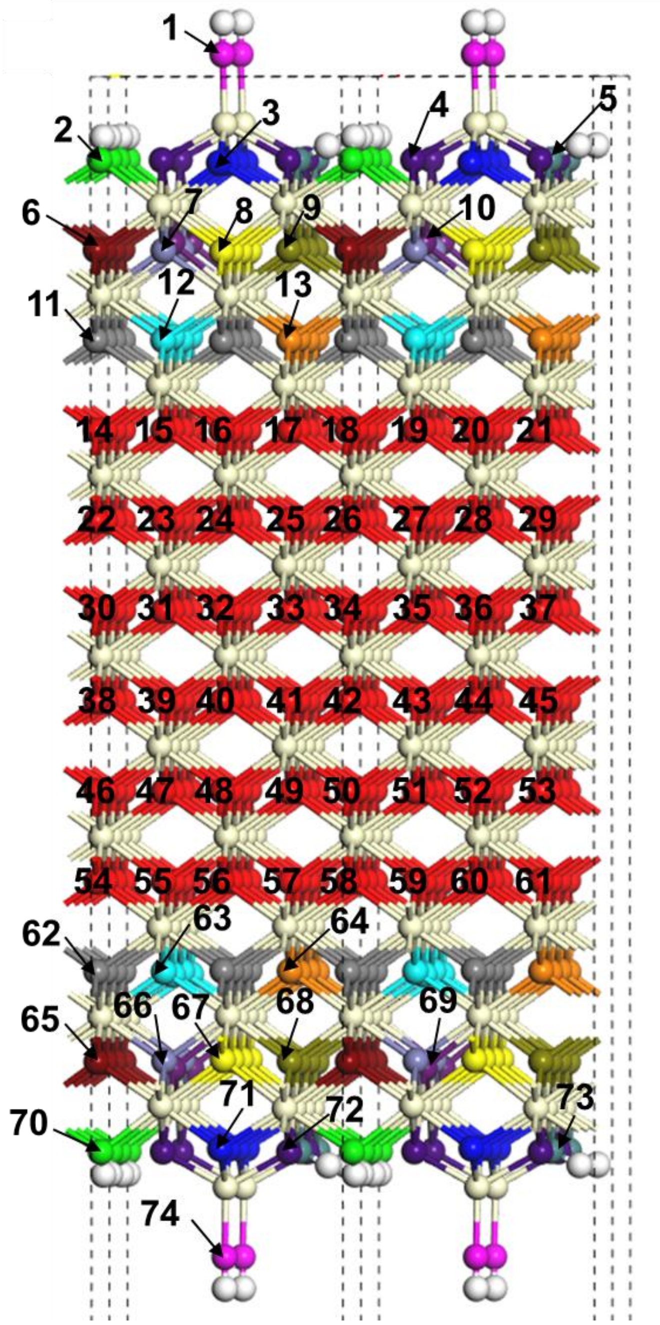

+

Supplementary Figure 35. Calculated structure of the hydrated CeO_4-t model with two H_2O molecules dissociatively adsorbed on each surface unit (M_2). Oxygen ions with the same chemical environment (thus the same NMR parameters) are labeled with the same number (Supplementary Table 12). Red, off-white and white spheres represent bulk oxygen, cerium and hydrogen ions, respectively. Surface oxygen groups with different chemical shifts are in different colors.

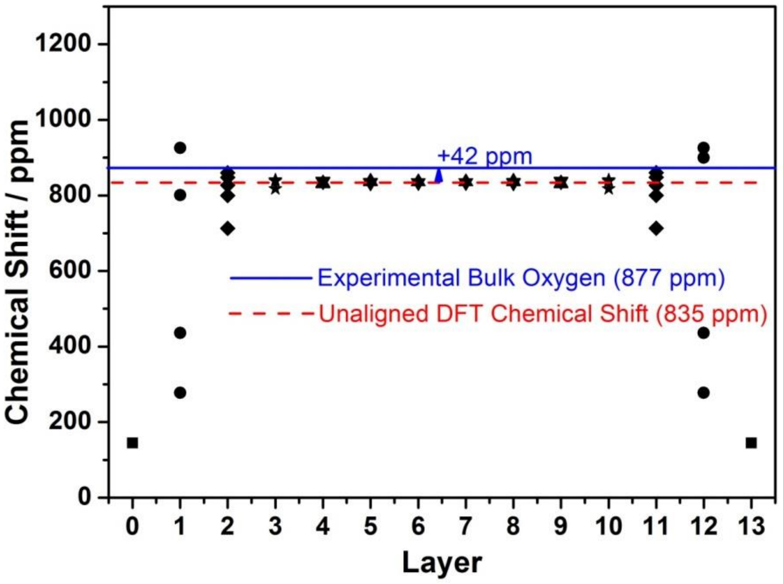

Supplementary Figure 36. The determination of the ^{17}O reference chemical shift (δ_{ref}) for M_2. Spheres, diamonds, stars and triangles (overlap from layer 4 to 9 because of the small numerical difference) represent the unaligned DFT chemical shifts of oxygen ions at the top/bottom 1st, 2nd and 3rd surface layers and the inner part of the structure ("bulk"). The red dashed line shows the average value of the unaligned DFT chemical shifts of the oxygen ions in the "bulk".

Supplementary Table 12. Calculated ^{17}O NMR parameters (isotropic chemical shifts (δ_{iso}), quadrupolar parameters (C_Q and η) and calculated NMR shifts (center of gravity, δ_{CG}s)) for oxygen ions in M_2. The corresponding structure is presented in Supplementary Figure 35.

O_{ion} No.	δ_{iso} / ppm	C_Q / MHz	η	δ_{CG} / ppm	Assignment
1	187	7.53	0.00	71	Surface -OH
2	320	6.88	0.18	222	Surface -OH
3	968	0.40	0.21	968	1st Layer O_{3C}
4	943	0.39	0.97	943	1st Layer O_{3C}
5	478	5.53	0.25	414	Surface -OH
6	890	0.22	0.37	890	2nd Layer O_{4C}
7	756	0.25	0.86	756	2nd Layer O_{4C}
8	869	0.19	0.55	869	2nd Layer O_{4C}
9	902	0.22	0.05	902	2nd Layer O_{4C}
10	842	0.62	0.33	841	2nd Layer O_{4C}
11	883	0.13	0.58	883	3rd Layer O_{4C}
12	880	0.26	0.75	880	3rd Layer O_{4C}
13	860	0.19	0.44	860	3rd Layer O_{4C}
14	884	0.11	0.26	884	Bulk Like
15	875	0.19	0.40	875	Bulk Like
16	884	0.08	0.77	884	Bulk Like
17	873	0.11	0.24	873	Bulk Like
18	881	0.11	0.26	881	Bulk Like
19	875	0.19	0.40	875	Bulk Like
20	881	0.08	0.77	881	Bulk Like
21	873	0.11	0.24	873	Bulk Like
22	878	0.22	0.35	878	Bulk Like
23	875	0.14	0.29	875	Bulk Like
24	880	0.10	0.23	880	Bulk Like
25	877	0.12	0.08	877	Bulk Like
26	878	0.22	0.35	878	Bulk Like
27	875	0.14	0.29	875	Bulk Like
28	880	0.10	0.23	880	Bulk Like
29	877	0.12	0.08	877	Bulk Like
30	878	0.11	0.03	878	Bulk Like
31	876	0.12	0.06	876	Bulk Like
32	878	0.10	0.04	878	Bulk Like
33	876	0.12	0.03	876	Bulk Like
34	878	0.11	0.03	878	Bulk Like
35	876	0.12	0.06	876	Bulk Like
36	878	0.10	0.04	878	Bulk Like
37	876	0.12	0.03	876	Bulk Like
38	878	0.11	0.08	878	Bulk Like
39	876	0.13	0.11	876	Bulk Like
40	878	0.10	0.10	878	Bulk Like
41	876	0.12	0.08	876	Bulk Like
42	878	0.11	0.08	878	Bulk Like
43	876	0.13	0.11	876	Bulk Like
44	878	0.10	0.10	878	Bulk Like
45	876	0.12	0.08	876	Bulk Like

46	878	0.11	0.17	878	Bulk Like
47	875	0.14	0.39	875	Bulk Like
48	880	0.10	0.28	880	Bulk Like
49	877	0.12	0.17	877	Bulk Like
50	878	0.11	0.17	878	Bulk Like
51	875	0.14	0.39	875	Bulk Like
52	880	0.10	0.28	880	Bulk Like
53	877	0.12	0.17	877	Bulk Like
54	884	0.11	0.34	884	Bulk Like
55	875	0.19	0.45	875	Bulk Like
56	884	0.08	0.88	884	Bulk Like
57	873	0.11	0.27	873	Bulk Like
58	881	0.11	0.34	881	Bulk Like
59	875	0.19	0.45	875	Bulk Like
60	881	0.08	0.88	881	Bulk Like
61	873	0.11	0.27	873	Bulk Like
62	883	0.13	0.61	883	10th Layer O_{4C}
63	880	0.25	0.80	880	10th Layer O_{4C}
64	860	0.19	0.46	860	10th Layer O_{4C}
65	890	0.21	0.35	890	11th Layer O_{4C}
66	756	0.25	0.82	756	11th Layer O_{4C}
67	869	0.19	0.50	869	11th Layer O_{4C}
68	902	0.22	0.06	902	11th Layer O_{4C}
69	842	0.63	0.34	841	11th Layer O_{4C}
70	320	6.68	0.18	222	Surface -OH
71	968	0.40	0.20	968	12th Layer O_{3C}
72	943	0.40	0.96	943	12th Layer O_{3C}
73	478	5.56	0.25	414	Surface -OH
74	187	7.53	0.00	71	Surface -OH

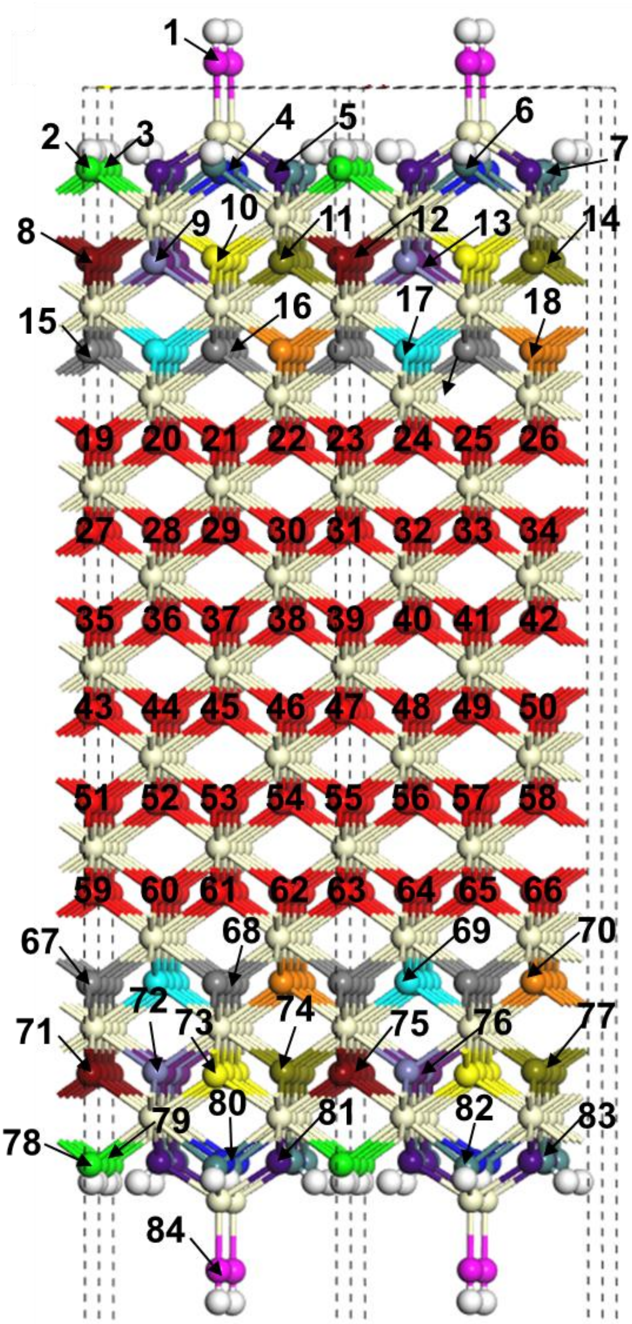

Supplementary Figure 37. The calculated structure of the hydrated CeO_4-t model with three H_2O molecules dissociatively adsorbed on each surface unit (M_3). Oxygen ions with the same chemical environment (thus the same NMR parameters) are labeled with the same number (Supplementary Table 13). Red, off-white and white spheres represent bulk oxygen, cerium and hydrogen ions, respectively. Surface oxygen groups with different chemical shifts are in different colors.

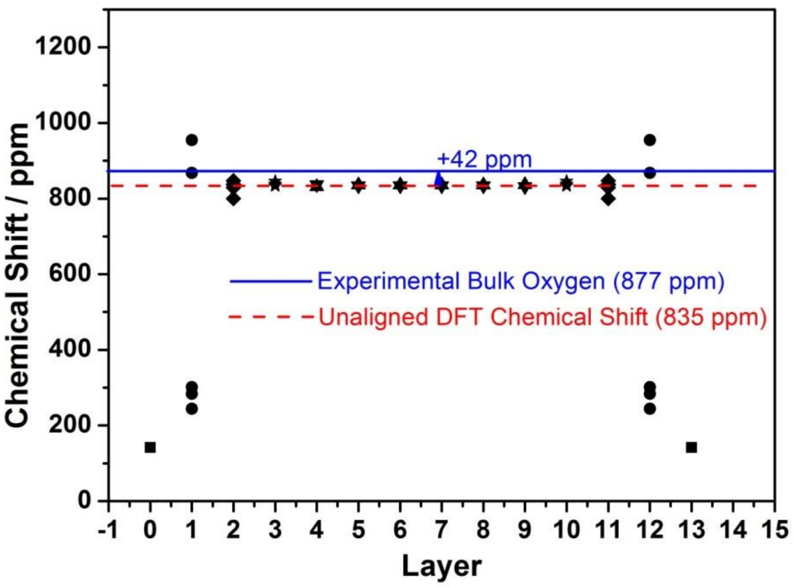

Supplementary Figure 38. The determination of the [17]O reference chemical shift (δ_{ref}) for M_3. Spheres, diamonds, stars and triangles (overlap from layer 4 to 9 because of the small numerical difference) represent the unaligned DFT chemical shifts of oxygen ions at the top/bottom 1st, 2nd and 3rd surface layers and the inner part of the structure (“bulk”). The red dashed line shows the average value of the unaligned DFT chemical shifts of the oxygen ions in the “bulk”.

Supplementary Table 13. Calculated ^{17}O NMR parameters (isotropic chemical shifts (δ_{iso}), quadrupolar parameters (C_Q and η) and calculated NMR shifts (center of gravity, δ_{CG}s)) for oxygen ions in M_3. The corresponding structure is presented in Supplementary Figure 37.

O_{ion} No.	δ_{iso} / ppm	C_Q / MHz	η	δ_{CG} / ppm	Assignment
1	184	7.52	0.01	69	Surface -OH
2	343	6.78	0.08	249	Surface -OH
3	326	6.85	0.12	230	Surface -OH
4	997	0.50	0.64	996	1st Layer O_{3C}
5	910	0.32	0.84	910	1st Layer O_{3C}
6	286	6.23	0.15	206	Surface -OH
7	385	6.74	0.19	291	Surface -OH
8	842	0.29	0.55	842	2nd Layer O_{4C}
9	879	0.13	0.69	879	2nd Layer O_{4C}
10	871	0.12	0.76	871	2nd Layer O_{4C}
11	880	0.13	0.61	880	2nd Layer O_{4C}
12	889	0.21	0.33	889	2nd Layer O_{4C}
13	887	0.20	0.71	887	2nd Layer O_{4C}
14	887	0.21	0.69	887	2nd Layer O_{4C}
15	876	0.12	0.35	876	3rd Layer O_{4C}
16	868	0.13	0.70	868	3rd Layer O_{4C}
17	880	0.10	0.65	880	3rd Layer O_{4C}
18	880	0.10	0.64	880	3rd Layer O_{4C}
19	874	0.11	0.16	874	Bulk Like
20	879	0.10	0.23	879	Bulk Like
21	874	0.11	0.30	874	Bulk Like
22	879	0.10	0.22	879	Bulk Like
23	874	0.11	0.16	874	Bulk Like
24	879	0.10	0.23	879	Bulk Like
25	874	0.11	0.30	874	Bulk Like
26	879	0.10	0.22	879	Bulk Like
27	876	0.11	0.04	876	Bulk Like
28	878	0.10	0.10	878	Bulk Like
29	876	0.11	0.04	876	Bulk Like
30	878	0.10	0.10	878	Bulk Like
31	876	0.11	0.04	876	Bulk Like
32	878	0.10	0.10	878	Bulk Like
33	876	0.11	0.04	876	Bulk Like
34	878	0.10	0.10	878	Bulk Like
35	876	0.11	0.01	876	Bulk Like
36	878	0.10	0.01	878	Bulk Like
37	876	0.11	0.01	876	Bulk Like
38	878	0.10	0.01	878	Bulk Like
39	876	0.11	0.01	876	Bulk Like
40	878	0.10	0.01	878	Bulk Like
41	876	0.11	0.01	876	Bulk Like
42	878	0.10	0.01	878	Bulk Like
43	876	0.11	0.01	876	Bulk Like
44	878	0.10	0.01	878	Bulk Like
45	876	0.11	0.01	876	Bulk Like

46	878	0.10	0.01	878	Bulk Like
47	876	0.11	0.01	876	Bulk Like
48	878	0.10	0.01	878	Bulk Like
49	876	0.11	0.01	876	Bulk Like
50	878	0.10	0.01	878	Bulk Like
51	876	0.11	0.04	876	Bulk Like
52	878	0.10	0.10	878	Bulk Like
53	876	0.11	0.04	876	Bulk Like
54	878	0.10	0.10	878	Bulk Like
55	876	0.11	0.04	876	Bulk Like
56	878	0.10	0.10	878	Bulk Like
57	876	0.11	0.04	876	Bulk Like
58	878	0.10	0.10	878	Bulk Like
59	874	0.11	0.16	874	Bulk Like
60	879	0.10	0.23	879	Bulk Like
61	874	0.11	0.30	874	Bulk Like
62	879	0.10	0.22	879	Bulk Like
63	874	0.11	0.16	874	Bulk Like
64	879	0.10	0.23	879	Bulk Like
65	874	0.11	0.30	874	Bulk Like
66	879	0.10	0.22	879	Bulk Like
67	876	0.12	0.33	876	10th Layer O_{4C}
68	868	0.14	0.72	868	10th Layer O_{4C}
69	880	0.10	0.59	880	10th Layer O_{4C}
70	880	0.10	0.64	880	10th Layer O_{4C}
71	842	0.29	0.56	842	11th Layer O_{4C}
72	879	0.13	0.66	879	11th Layer O_{4C}
73	871	0.12	0.76	871	11th Layer O_{4C}
74	880	0.13	0.59	880	11th Layer O_{4C}
75	889	0.21	0.37	889	11th Layer O_{4C}
76	887	0.20	0.72	887	11th Layer O_{4C}
77	887	0.21	0.66	887	11th Layer O_{4C}
78	343	6.68	0.01	249	Surface -OH
79	326	6.85	0.12	230	Surface -OH
80	997	0.49	0.63	996	12th Layer O_{3C}
81	910	0.32	0.88	910	12th Layer O_{3C}
82	286	6.23	0.15	206	Surface -OH
83	385	6.73	0.19	291	Surface -OH
84	184	6.75	0.18	69	Surface -OH

Supplementary Note 2

Surface free energies of different structures including the O-t model with 0, 1, 2, 3, and 4 dissociative water molecules per surface unit and the CeO_4-t model with 0, 1, 2, and 3 dissociative water molecules per surface unit (M_0, M_1, M_2, and M_3) were calculated in Supplementary Figure 39 to indicate the relative stabilities of these structures at experimental conditions. Considering that surface reconstructions generally occur at harsh conditions,[9] the ceria (100) surface structure should form during the annealing procedure before ^{17}O isotropic labeling (at 573 K with a water pressure between 1 and 1000 Pa) and remain unchanged by the ^{17}O isotopic labeling process and NMR measurements at relatively mild conditions (a temperature lower than 573 K and water pressure higher than 1000 Pa). Supplementary Figure 39 shows that M_1 is the most stable model at 573 K under water pressures ranging from 1 to 1000 Pa. This further suggests the occurrence of CeO_4 reconstructions on the ceria (100) surface before ^{17}O enrichment. The following isotopic labeling process causes different degrees of ^{17}O exchange and an increase of water coverage, resulting in the presence of corresponding surface structures such as M_2 and M_3 in NCs-$^{17}O_2$ and NCs-$H_2{}^{17}O$, respectively.

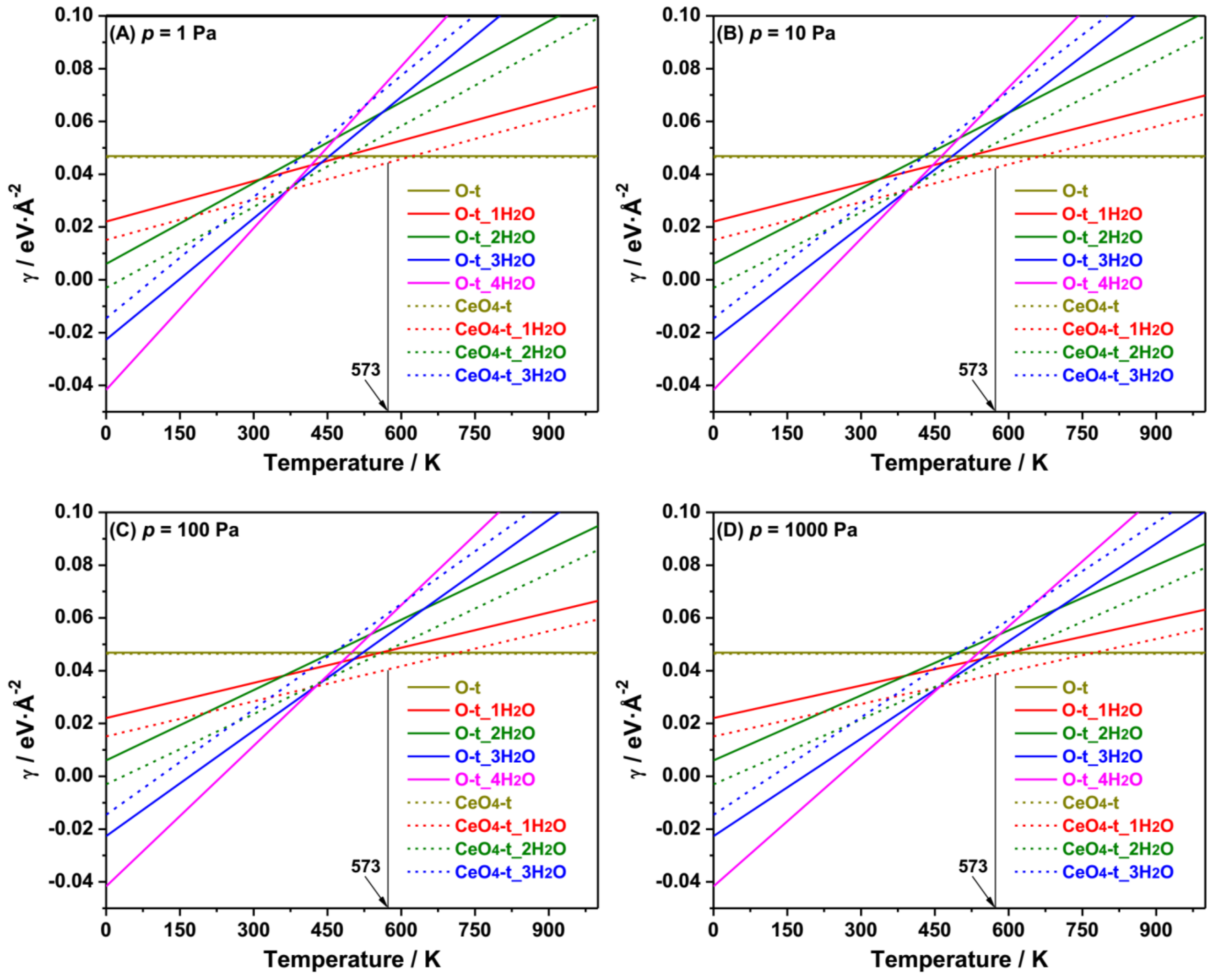

Supplementary Figure 39. Calculated surface free energies (γ) of different structures as a function of temperature (T) and water pressures (p). (A) 1, (B) 10, (C) 100, and (D) 1000 Pa.

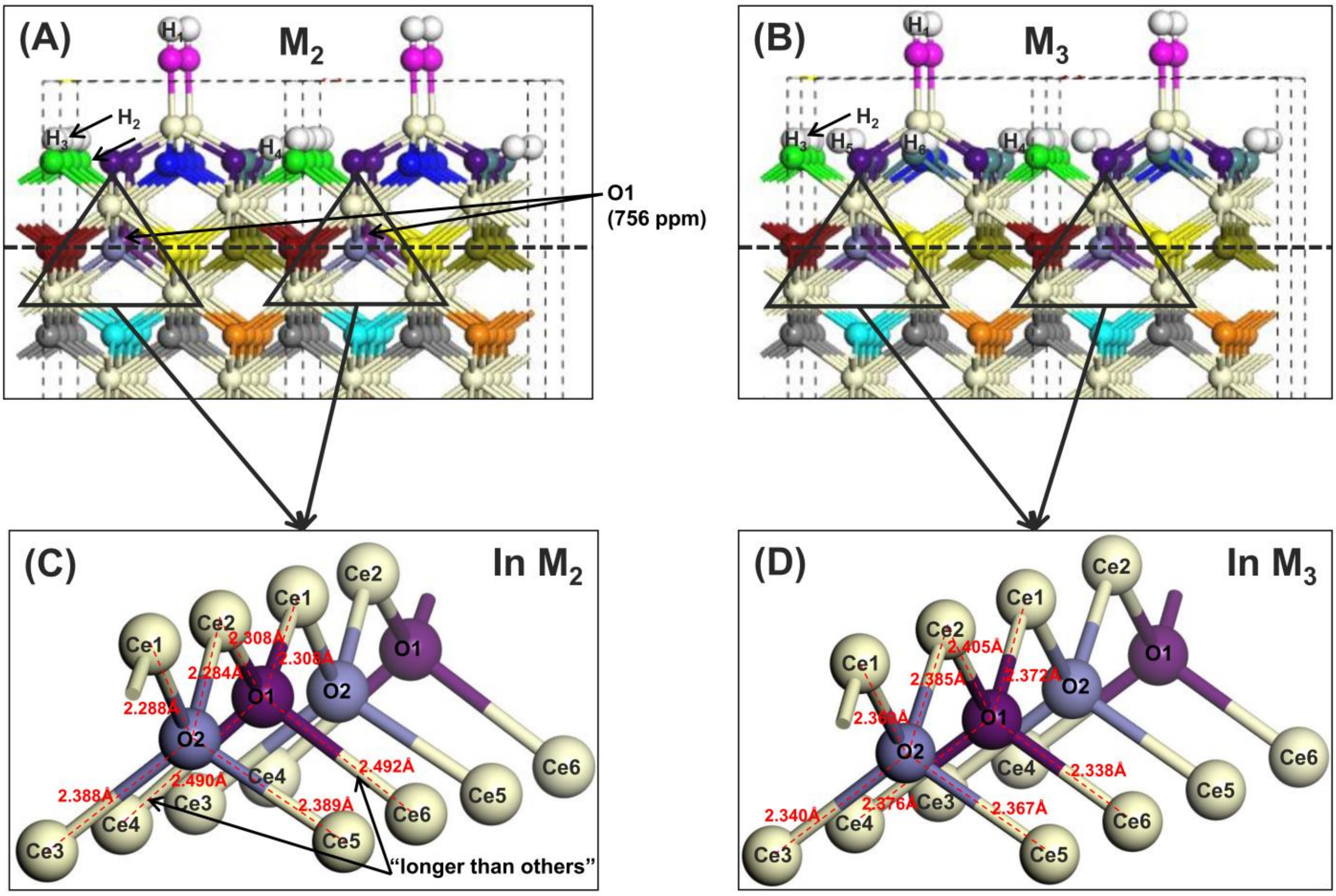

Supplementary Figure 40. Calculated surface structure of M_2 and M_3 as well as enlargements of the second layer . (A, C) M_2 and (B, D) M_3. Oxygen ions with the same chemical environment (thus possessing the same NMR parameters) are labeled with the same number. Off-white and white spheres respectively represent cerium and hydrogen ions. In figure C and D, the cerium–oxygen bond lengths are presented. The "O1" ions, which are calculated to have a chemical shift of 756 ppm, have longer cerium–oxygen bond lengths according to the DFT calculation.

Section F: The determination of the ^{1}H δ_{ref} for M_2 and M_3

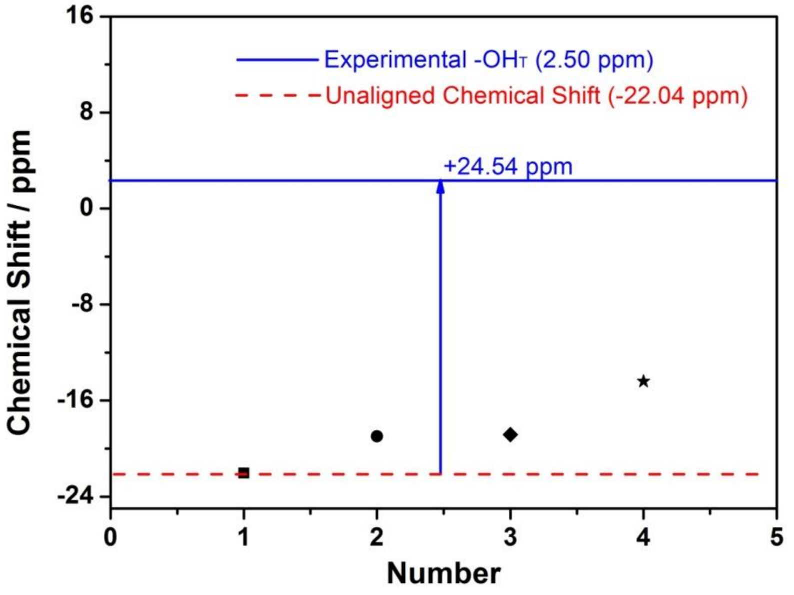

Supplementary Figure 41. The determination of the reference ^{1}H chemical shift (δ_{ref}) for M_2. The square, sphere, diamond and star represent the unaligned DFT chemical shifts of hydrogen ions on the surface.

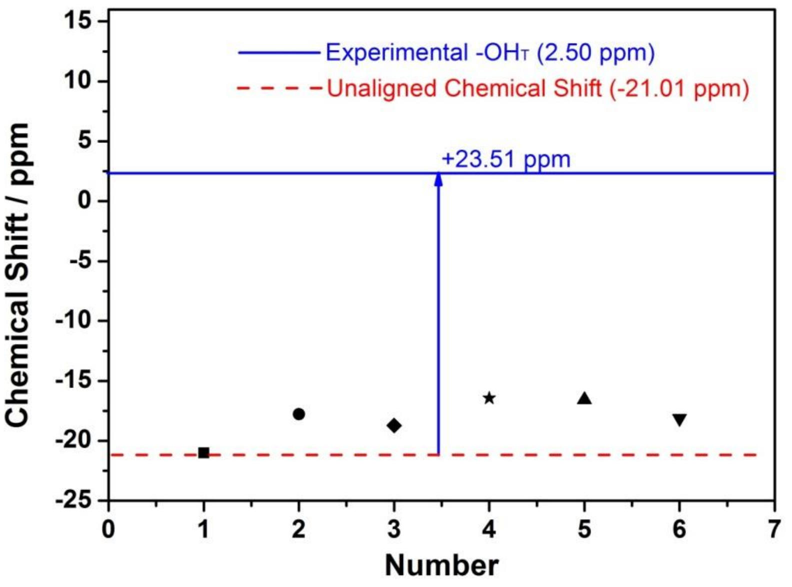

Supplementary Figure 42. The determination of the reference ^{1}H chemical shift (δ_{ref}) for M_3. The square, sphere, diamond and star represent the unaligned DFT chemical shifts of hydrogen ions on the surface.

Section G: ^{17}O direct DNP MATPASS NMR spectrum of NCs-$H_2{}^{17}O$

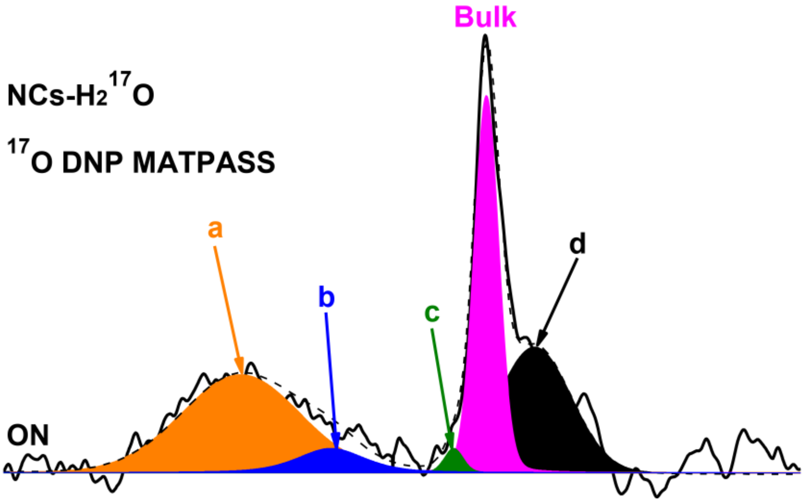

Supplementary Figure 43. The isotropic slice of the ^{17}O direct DNP projection magic angle turning and phase adjusted sideband separation (MATPASS) NMR spectrum of NCs-$H_2{}^{17}O$ with microwave irradiation on. The recycle delay was 60 s.[10,11]

Supplementary References

1. Shyu, J. Z., Weber, W. H. & Gandhi, H. S. Surface characterization of alumina-supported ceria. *J. Phys. Chem.* **92**, 4964–4970 (1988).
2. McBride, J. R. *et al.* Raman and X−ray studies of $Ce_{1-x}RE_xO_{2-y}$, where RE=La, Pr, Nd, Eu, Gd, and Tb. *J. Appl. Phys.* **76**, 2435–2441 (1994).
3. Meng, L. *et al.* Synergetic effects of PdO species on CO oxidation over PdO-CeO_2 catalysts. *J. Phys. Chem. C* **115**, 19789–19796 (2011).
4. Wang, Y. *et al.* Heterogeneous ceria catalyst with water-tolerant Lewis acidic sites for one-pot synthesis of 1,3-diols via prins condensation and hydrolysis reactions. *J. Am. Chem. Soc.* **135**, 1506–1515 (2013).
5. Wang, M. *et al.* Identification of different oxygen species in oxide nanostructures with ^{17}O solid-state NMR spectroscopy. *Sci. Adv.* **1**, e1400133 (2015).
6. Li, Y. *et al.* Distinguishing faceted oxide nanocrystals with ^{17}O solid-state NMR spectroscopy. *Nat. Commun.* **8**, 581 (2017).
7. Lippmaa, E., Samoson, A. & Magi, M. High-resolution ^{27}Al NMR of aluminosilicates. *J. Am. Chem. Soc.* **108**, 1730–1735 (1986).
8. Kropp, T., Paier, J. & Sauer, J. Interactions of water with the (111) and (100) surfaces of ceria. *J. Phys. Chem. C* **121**, 21571–21578 (2017).
9. Pan, Y. *et al.* Ceria nanocrystals exposing wide (100) facets: Structure and polarity compensation. *Adv. Mater. Interfaces* **1**, 1400404 (2014).
10. Halat, D. M. *et al.* Probing oxide-ion mobility in the mixed ionic-electronic conductor $La_2NiO_{4+\delta}$ by solid-state ^{17}O MAS NMR spectroscopy. *J. Am. Chem. Soc.* **138**, 11958–11969 (2016).
11. Bielecki, A. & Burum, D. P. Temperature dependence of ^{207}Pb MAS spectra of solid lead nitrate. An accurate, sensitive thermometer for variable-temperature MAS. *J. Magn. Reson.* **116**, 215–220 (1995).
